# Unraveling Melanin Biosynthesis and Signaling Networks in Cryptococcus neoformans

**DOI:** 10.1128/mBio.02267-19

**Published:** 2019-10-01

**Authors:** Dongpil Lee, Eun-Ha Jang, Minjae Lee, Sun-Woo Kim, Yeonseon Lee, Kyung-Tae Lee, Yong-Sun Bahn

**Affiliations:** a Department of Biotechnology, College of Life Science and Biotechnology, Yonsei University, Seoul, Republic of Korea; University of Texas Health Science Center

**Keywords:** Bzp4, Usv101, Mbs1, Hob1, Gsk3, TOR pathway, RAM pathway, Bzp4

## Abstract

Melanin is an antioxidant polyphenol pigment required for the pathogenicity of many fungal pathogens, but comprehensive regulatory mechanisms remain unidentified. In this study, we systematically analyzed melanin-regulating signaling pathways in Cryptococcus neoformans and identified four melanin-regulating core transcription factors (TFs), Bzp4, Usv101, Mbs1, and Hob1, required for induction of the laccase gene (*LAC1*). Bzp4, Usv101, and Mbs1 independently regulate *LAC1* induction, whereas Hob1 controls Bzp4 and Usv101 expression. Both Bzp4 and Usv101 are localized in the cytoplasm under nutrient-rich conditions (i.e., in the presence of yeast extract-peptone-dextrose [YPD] medium) but translocate into the nucleus upon nutrient starvation (i.e., in the presence of yeast nitrogen base [YNB] medium without glucose), and Mbs1 is constitutively localized in the nucleus. Notably, the cAMP pathway is not involved in regulation of the four TFs, but the high-osmolarity glycerol response (HOG) pathway negatively regulates induction of *BZP4* and *LAC1*. Next, we searched for potential kinases upstream of the core TFs and identified nine core kinases; their deletion led to defective melanin production and *LAC1* induction. Deletion of *GSK3* or *KIC1* abolished induction of *LAC1* and *BZP4* and perturbed nuclear translocation of Bzp4. Notably, Gsk3 also regulated expression of *HOB1*, *USV101*, and *MBS1*, indicating that it is a critical melanin-regulating kinase. Finally, an RNA sequencing-based transcriptome analysis of the wild-type strain and of *bzp4*Δ, *usv101*Δ, *hob1*Δ, and *mbs1*Δ strains under nutrient-rich and nutrient-starved conditions revealed that the melanin-regulating core TFs govern redundant and distinct classes of genes involved in a variety of biological processes.

## Abstract (precis)

Melanins are dark green, brown, or black pigments that serve as antioxidant, reactive oxygen species (ROS) scavengers that protect fungal pathogens from radiation and host immune responses. Cryptococcus neoformans, the major etiological agent of fungal meningoencephalitis, also utilizes melanin as a key virulence factor. In this basidiomycete pathogen, melanin production is regulated by the cAMP and high-osmolarity glycerol response (HOG) pathways, and yet its complex signaling networks remain poorly described. In this study, we uncovered novel melanin synthesis regulatory networks consisting of core transcription factors (TFs), including Bzp4, Usv101, Hob1, and Mbs1, and core kinases Gsk3 and Kic1. These networks were identified through coupling systematic analyses of the expression and epistatic relationships of TF and kinase mutant libraries in the presence of diverse melanin substrates with transcriptome profiling of the core TF mutants. Thus, this report provides comprehensive insight into the melanin-regulating pathways in C. neoformans and other fungal pathogens.

## INTRODUCTION

Melanins are highly ordered polyphenolic and/or polyindolic biological pigments found in diverse living organisms, including animals, fungi, and bacteria (1–3). Melanins have high molecular masses, negative charges, and hydrophobicity (4–8). Due to their chemical complexity and insolubility in aqueous or organic solvents, the exact melanin structure remains unsolved, but recent advanced nuclear magnetic resonance (NMR) and electron microscopy technologies have provided glimpses of the amorphous melanin structure (9). These physicochemical characteristics of melanin impart features that mediate various cellular functions, such as thermotolerance and reactive oxygen species (ROS) resistance (10–12), enabling organisms to adapt to diverse environmental conditions.

Melanins are present in a number of pathogenic fungi as three principal types: 1,8-dihydroxynaphthalene (DHN) melanin, 3,4-dihydroxyphenylalanine (DOPA)-melanin (eumelanin), and pyomelanin (13, 14). DHN melanin is synthesized from acetyl-coenzyme A via the polyketide synthase pathway. The filamentous fungus Aspergillus fumigatus produces DHN melanin, which is responsible for the gray-green color of its conidia, and deletion of the polyketide synthase PksP results in white spores and attenuated virulence (15). Eumelanin is catalyzed by a polyphenol oxidase (laccase) using exogenous *o-*diphenolic or *p-*diphenolic substrates. In Candida albicans, eumelanin particles are observed *in vitro* and in infected murine kidney and human skin tissues (16). In this ascomycete pathogen, melanin is externalized in the form of electron-dense melanosomes and extracellularly secreted or loosely bound to the cell wall surface through association with chitins (17). Although C. albicans has laccase activity (16), no candidate laccase gene has been discovered in its genome and the role of melanin in its pathogenicity remains unclear. Pyomelanin is an extracellular water-soluble pigment, which is in stark contrast to the cell wall-immobilized melanins DHN and DOPA (14). Pyomelanin is produced by the polymerization of homogentisic acid, one of the degradation products of l-tyrosine/l-phenylalanine. In A. fumigatus, pyomelanin is involved in the germination of conidia and in defense against external oxidants (14). The dimorphic human fungal pathogen Histoplasma capsulatum produces all three types of melanins (18, 19). Melanins are also involved in the virulence of plant-pathogenic fungi, including Magnaporthe grisea and Colletotrichum lagenarium (13, 20).

Melanin is a critical virulence factor in the basidiomycete fungal pathogen Cryptococcus neoformans, which causes fatal meningoencephalitis in immunocompromised patients and is responsible for more than 220,000 infections and 180,000 deaths globally every year (21, 22). In the presence of exogenous diphenolic compounds such as l-3,4-dihydroxyphenylalanine (l-DOPA), C. neoformans produces brown-colored eumelanin via laccases (Lac1 and Lac2) (22). Laccase is minimally expressed under nutrient-rich conditions, but expression is induced by nutrient starvation (23, 24). Once expressed, laccases are loaded into secretory vesicles and deposited as spherical particles within the cell wall by the use of chitin as an anchoring molecule or are secreted extracellularly (25–29). Furthermore, melanins promote survival in the environment and within hosts, protecting C. neoformans from UV or extreme ionizing irradiation, oxidative damage, and extreme temperatures (11, 12, 30, 31) and from macrophage phagocytosis during infection (32). Even after phagocytosis, melanized cells are resistant to ROS and microbicidal peptides produced by macrophages (11, 33). Therefore, mutants lacking the melanin pigment lose virulence (10, 34–36). Notably, the neurotropism of C. neoformans is partially attributable to its ability to convert catecholamine neurotransmitters, including dopamine, norepinephrine, and epinephrine, into melanin (22, 37).

Due to the clinical importance of melanins in the pathogenicity of C. neoformans, intensive efforts have been made to elucidate the signaling networks governing its biosynthesis. Prior studies have revealed two major signaling pathways, namely, the cyclic AMP/protein kinase A (cAMP/PKA) and high-osmolarity glycerol response (HOG) pathways. Whereas perturbation of the cAMP/PKA pathway significantly reduces *LAC1* induction and melanin production (38–40), inhibition of the HOG pathway increases melanin production and restores normal melanin production in cAMP mutants (41, 42). Systematic functional analyses of C. neoformans transcription factors (TFs) and kinases have revealed signaling components potentially involved in melanin production (43, 44), but a comprehensive understanding of melanin-regulating signaling networks is far from completion. In this study, we systematically analyzed melanin-regulating signaling networks in C. neoformans using TF and kinase mutant libraries that we had previously constructed. Here, we describe the discovery of four melanin-regulating core TFs, Bzp4, Hob1, Usv101, and Mbs1, and elucidate their upstream kinases, Gsk3, Kic1, and Pkh202, and downstream signaling regulators and effectors. Through this report, we provide further insights into the complex regulatory networks of melanin biosynthesis in C. neoformans.

## RESULTS

### Bzp4, Hob1, Usv101, and Mbs1 are melanin-regulating core transcription factors in C. neoformans.

**FIG 1:**
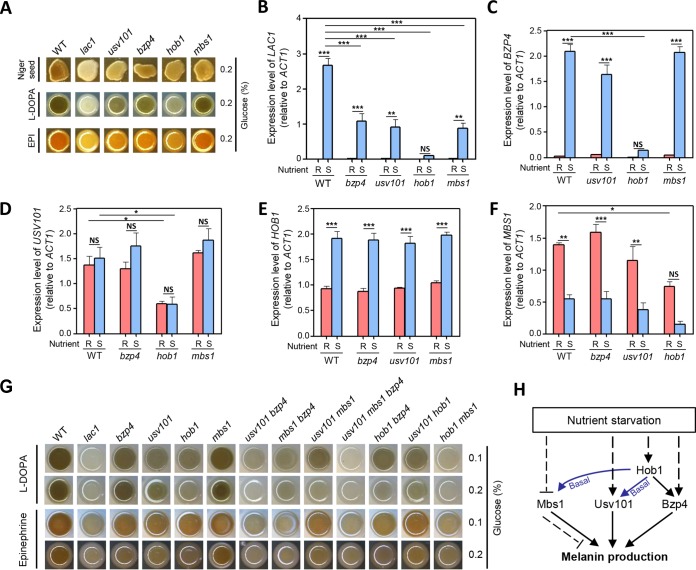
Melanin-regulating core transcription factors in C. neoformans. (A) C. neoformans WT and mutant strains on Niger seed, dopamine, and epinephrine medium. Darker cultures had more effective melanin synthesis than those with lighter colors. (B to F) Quantitative reverse transcription-PCR (RT-PCR) was performed using total RNA of each strain under nutrient-rich (R; YPD) or nutrient-starved (S; YNB without glucose) conditions. The induction of (B) *LAC1*, (C) *BZP4*, (D) *USV101*, (E) *HOB1*, and (F) *MBS1* by nutrient starvation was measured in WT and mutant strains. Three biologically independent experiments were performed with three technical replicates each. Error bars indicate standard errors of the means (SEM). Statistical differences among gene expression levels were calculated by one-way analysis of variance (ANOVA) multiple comparisons performed with Bonferroni’s correction (*, *P < *0.05; **, *P < *0.01; ***, *P < *0.001; NS, not significant). (G) Each strain was spotted on medium plates containing dopamine and epinephrine. Darker cultures had more effective melanin synthesis than those with lighter colors. (H) Proposed regulatory relationships among Hob1, Mbs1, Usv101, and Bzp4 for melanin production.

We previously reported 27 TFs (11 positive regulators and 16 negative regulators) that are involved in melanin production on Niger seed medium (43). For the corresponding TF deletion mutants, we reevaluated their melanin production levels on other melanin-inducing media containing l-DOPA or epinephrine (See Fig. S1 in the supplemental material). Among these, the deletion of four TFs (i.e., *MBS1*, *BZP4*, *USV101*, and *HOB1*) resulted in defective melanin production on all three melanin-inducing media (Fig. 1A). The *bzp4*Δ and *hob1*Δ mutants, which are the TF mutants most defective in melanin production on Niger seed medium (43), showed the most marked reduction in melanin synthesis on l-DOPA and epinephrine media. The *usv101*Δ and *mbs1*Δ mutants exhibited weakly reduced melanin production in all three media. Notably, however, the *cuf1*Δ and *fzc8*Δ mutants, which are highly defective in melanin production on Niger seed medium (43), did not exhibit significantly altered melanin production on l-DOPA and epinephrine media. Surprisingly, the *yap1*Δ, *ada2*Δ, and *gat1*Δ mutants, which have increased melanin levels on Niger seed medium (43), showed reduced melanin production on l-DOPA and epinephrine media. The remaining TF mutants did not show altered melanin production on l-DOPA and epinephrine media. Together, these results indicate that signaling pathways involved in melanin biosynthesis could be differentially regulated depending on medium conditions but that Bzp4, Hob1, Usv101, and Mbs1 appear to be melanin-regulating core TFs regardless of the type of melanin-inducing media. However, none of them exhibited the complete lack of melanin production observed in the *lac1*Δ mutant, indicating that multiple TFs may cooperate to control melanin production.

10.1128/mBio.02267-19.1 FIG S1 Melanin production levels on media containing l-DOPA and epinephrine for the previously reported 27 transcription factor mutants showing altered levels of melanin production on Niger seed medium. The WT strain, the *cac1*Δ (YSB42) mutant, and the 27 TF mutants were spotted on medium plates containing dopamine and epinephrine (EPI). The 27 transcription factor mutants were previously reported to show altered melanin production levels on Niger seed medium (43). Darker cultures had more effective melanin synthesis than those with lighter colors. Download FIG S1, PDF file, 1.2 MB. Copyright © 2019 Lee et al. 2019 Lee et al. This content is distributed under the terms of the Creative Commons Attribution 4.0 International license.

Because *LAC1* expression is induced by nutrient starvation (45), we addressed whether Mbs1, Bzp4, Usv101, and Hob1 control *LAC1* induction upon shifting from nutrient-rich conditions (yeast extract-peptone-dextrose [YPD]) to nutrient-starved conditions (yeast nitrogen base [YNB] without glucose). *LAC1* induction mediated by nutrient starvation was significantly reduced in the *bzp4*Δ, *usv101*Δ, *hob1*Δ, and *mbs1*Δ mutants compared to the wild-type (WT) strain (Fig. 1B). Among these, *HOB1* deletion resulted in the most severe defects in *LAC1* induction (Fig. 1B), which supported the finding that melanin synthesis was more severely reduced in the *hob1*Δ mutant than in the other TF mutants (Fig. 1A). These results indicate that the four TFs may cooperate to promote *LAC1* induction under nutrient starvation conditions, although Hob1 appears to play a dominant role. We then examined whether expression of *BZP4*, *USV101*, *HOB1*, and *MBS1* was also induced by nutrient starvation. Expression of *BZP4* and *HOB1*, but not *USV101*, was strongly induced by nutrient starvation whereas *MBS1* expression was significantly reduced (Fig. 1C to F). Notably, *BZP4* was expressed at very low levels under nutrient-rich conditions, but its expression was strongly induced by nutrient starvation (Fig. 1C), a pattern very similar to that seen with *LAC1* (Fig. 1B).

To elucidate the potential epistatic relationships among *BZP4*, *USV101*, *HOB1*, and *MBS1* with respect to melanin production, we monitored their expression patterns in each deletion mutant under nutrient-rich and nutrient-starved conditions. As seen with the wild-type strain, strong *BZP4* induction mediated by nutrient starvation was observed in *usv101*Δ and *mbs1*Δ mutants but not in the *hob1*Δ mutant (Fig. 1C), indicating that Hob1 governs *BZP4* induction under nutrient starvation conditions. In contrast, the level of *HOB1* induction mediated by nutrient starvation observed in the *bzp4*Δ, *usv101*Δ, and *mbs1*Δ mutants was similar to that seen in the wild-type strain (Fig. 1E). Notably, basal expression levels of *USV101* were significantly reduced in the *hob1*Δ mutant (Fig. 1D). These results strongly suggest that Hob1 positively regulates *BZP4* induction under conditions of nutrient starvation and basal expression levels of *USV101*. Reduced *MBS1* expression mediated by nutrient starvation similarly occurred in *bzp4*Δ, *usv101*Δ, and *hob1*Δ mutants, although basal *MBS1* expression levels were significantly lower in the *hob1*Δ mutant than in the wild-type strain and the other mutant strains (Fig. 1F).

To further elucidate the regulatory relationships among Bzp4, Hob1, Usv101, and Mbs1, we constructed a series of double-deletion mutants, including *usv101*Δ *bzp4*Δ, *mbs1*Δ *bzp4*Δ, *usv101*Δ *mbs1*Δ, *hob1*Δ *bzp4*Δ, *usv101*Δ *hob1*Δ, and *hob1*Δ *mbs1*Δ mutants (see Fig. S2 in the supplemental material). Supporting the expression patterns of *BZP4* and *USV101*, the *usv101*Δ *bzp4*Δ and *mbs1*Δ *bzp4*Δ double mutants exhibited more severe melanin defects than any single-mutation strain (Fig. 1G). These data suggest that Bzp4, Usv101, and Mbs1 play independent roles in melanin production. To further support the idea of this independence, we constructed a *usv101*Δ *mbs1*Δ *bzp4*Δ triple mutant. This triple-deletion mutant was even more defective in melanin production than each double mutant and was almost as defective as the *lac1*Δ mutant (Fig. 1G). Supporting the finding that Hob1 regulated basal *USV101* expression and *BZP4* induction by mediating nutrient starvation, the *hob1*Δ *mbs1*Δ mutant was highly defective in melanin production, albeit not at the level of the *usv101*Δ *mbs1*Δ *bzp4*Δ mutant (Fig. 1G). However, the *usv101*Δ *bzp4*Δ mutant was more defective in melanin synthesis than the *hob1*Δ mutant (Fig. 1G), suggesting that other regulators may also control Bzp4 and Usv101 expression or posttranslational modification. Collectively, these results demonstrate that Mbs1, Usv101, and Bzp4 are three major TFs that independently contribute to melanin production and that Hob1 regulates induction of *BZP4* by nutrient starvation and basal expression of Mbs1 and Usv101, although the latter two TFs appear to be transcriptionally and/or posttranslationally regulated by other unknown factors.

10.1128/mBio.02267-19.2 FIG S2 Southern blotting of gene deletion mutants constructed in this study. (A) *BZP4* was disrupted by a deletion cassette with the *NEO*^r^ marker in the *hob1*Δ, *usv101*Δ, and *mbs1*Δ mutant backgrounds. XbaI-digested genomic DNA of WT and *bzp4*Δ strains was used for Southern blotting. (B) *MBS1* was disrupted by a deletion cassette with the *NEO*^r^ marker in the *hob1*Δ mutant background or with the *HYG*^r^ marker in *usv101*Δ *bzp4*Δ and *usv101*Δ *mbs1*Δ mutant backgrounds. XhoI-digested genomic DNA of WT and *mbs1*Δ strains was used for Southern blotting. (C) *HOB1* was disrupted by a deletion cassette with the *NEO*^r^ marker in the *usv101*Δ mutant background. HincII-digested genomic DNA of WT and *hob1*Δ strains was used for Southern blotting. (D) *PKA1* was disrupted by a deletion cassette with the *HYG*^r^ marker in the *mbs1*Δ::*MBS1-mCherry* mutant background. PstI-digested genomic DNA of WT and *mbs1*Δ strains was used for Southern blotting. (E) *KIC1* was disrupted by a deletion cassette with the *HYG*^r^ marker in the *bzp4*Δ::*BZP4-mCherry* mutant background. PstI-digested genomic DNA of WT and *kic1*Δ strains was used for Southern blotting. (F) *GSK3* was disrupted by a deletion cassette with the *HYG*^r^ marker in the *bzp4*Δ::*BZP4-mCherry* mutant background. PstI-digested genomic DNA of WT and *gsk3*Δ strains was used for Southern blotting. (G) *PKA1* was disrupted by a deletion cassette with the *HYG*^r^ marker in the *mbs1*Δ::*MBS1-mCherry* mutant background. PstI-digested genomic DNA of WT and *pka1*Δ strains was used for Southern blotting. (H) *PKH202* was disrupted by a deletion cassette with the *HYG*^r^ marker in the *mbs1*Δ::*MBS1-mCherry* mutant background. XhoI-KpnI-digested genomic DNA of WT and *pkh202*Δ strains was used for Southern blotting. (I) *VPS30* was disrupted by a deletion cassette with the *NAT*^r^ marker in the WT background. ClaI/SphI-digested genomic DNA of WT and *vps30*Δ strains was used for Southern blotting. (J) *VPS34* was disrupted by a deletion cassette with the *NAT*^r^ marker in the WT background. ClaI-digested genomic DNA of WT and *vps34*Δ strains was used for Southern blotting. (K) CNAG_07029 was disrupted by a deletion cassette with the *NAT*^r^ marker in the WT background. SphI-digested genomic DNA of WT and *CNAG_07029*Δ strains was used for Southern blotting. Download FIG S2, PDF file, 0.9 MB. Copyright © 2019 Lee et al. 2019 Lee et al. This content is distributed under the terms of the Creative Commons Attribution 4.0 International license.

### The role of the cAMP/PKA and HOG pathways in regulation of Bzp4, Usv101, Hob1, and Mbs1.

**FIG 2:**
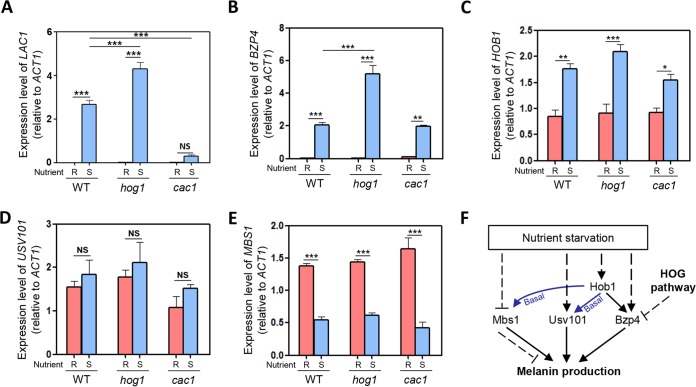
The role of cAMP/PKA and HOG pathways in regulating expression of *BZP4*, *USV101*, *HOB1*, and *MBS1*. (A to E) Quantitative RT-PCR was performed using total RNA of each strain under nutrient-rich (R; YPD) or nutrient-starved (S; YNB without glucose) conditions. The induction of (A) *LAC1*, (B) *BZP4*, (C) *HOB1*, (D) *USV101*, and (E) *MBS1* by nutrient starvation was measured in the WT and mutants. Three biologically independent experiments were performed with three technical replicates each. Error bars indicate standard errors of the means (SEM). Statistical differences among gene expression levels were calculated by one-way ANOVA multiple comparisons with Bonferroni’s correction (*, *P < *0.05; **, *P < *0.01; ***, *P < *0.001; NS, not significant). (F) Proposed regulatory relationship between Hob1, Mbs1, Usv101, Bzp4, and HOG pathways in melanin production.

A subsequent issue was which upstream signaling pathway(s) regulates these four TFs for melanin production. We predicted that the cAMP/PKA pathway was the most likely candidate for the following two reasons. First, deletion of key components in this pathway, such as adenylyl cyclase (Cac1) and the catalytic subunit of PKA (Pka1), severely abolishes melanin production (40). Second, *LAC1* induction mediated by nutrient starvation was found to be almost absent in the *cac1*Δ mutant (40) (Fig. 2A). Therefore, we examined whether the cAMP/PKA pathway regulates expression of these TFs under nutrient starvation conditions. Notably, we found that deletion of *CAC1* did not affect induction of *BZP4* (Fig. 2B), indicating that the cAMP/PKA pathway is dispensable, at least for induction of *BZP4*. Interestingly, *BZP4* was more strongly induced in the *hog1*Δ mutant than in the wild-type strain (Fig. 2B). In contrast, *HOB1*, *USV101*, and *MBS1* expression levels and patterns were not significantly affected by deletion of *CAC1* or *HOG1* (Fig. 2C to E). These data collectively implied that the cAMP/PKA pathway was not involved in transcriptional regulation of Hob1, Mbs1, Usv101, and Bzp4 and that the HOG pathway negatively regulated induction of Bzp4 by nutrient starvation (Fig. 2F). However, it is still possible that the cAMP pathway regulates these TFs by posttranslational modification such as phosphorylation or protein stability.

### Cellular localization of melanin-regulating core transcription factors.

**FIG 3:**
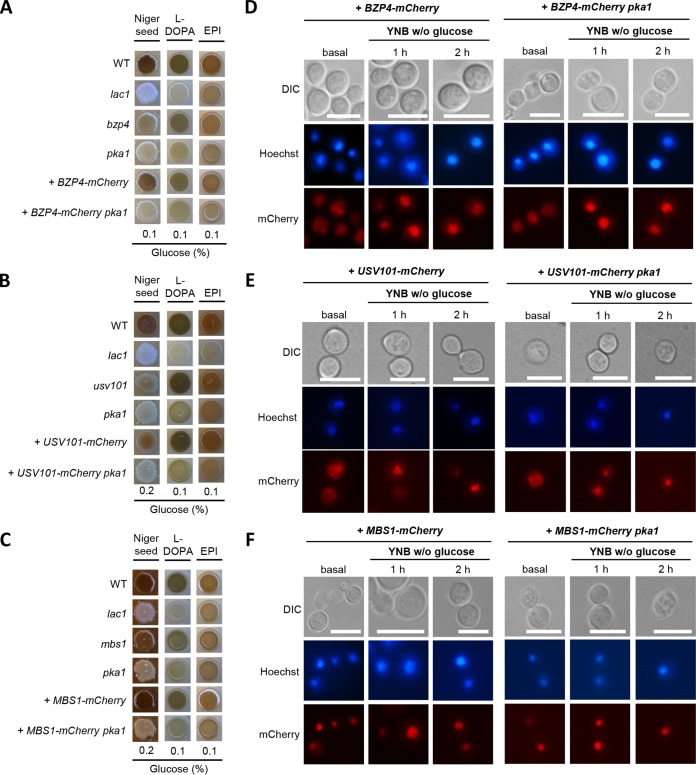
Cellular localization of the melanin-regulating core TFs in C. neoformans. (A to C) C. neoformans WT, *lac1*Δ, and *pka1*Δ with (A) *bzp4*Δ, *bzp4*Δ::*BZP4-mCherry*, *bzp4*Δ::*BZP4-mCherry pka1*Δ, (B) *usv101*Δ, *usv101*Δ::*USV101-mCherry*, *usv101*Δ::*USV101-mCherry pka1*Δ, and (C) *mbs1*Δ, *mbs1*Δ::*MBS1-mCherry*, *mbs1*Δ::*MBS1-mCherry pka1*Δ mutant strains on Niger seed, dopamine, and epinephrine medium. Darker cultures had more effective melanin synthesis than those with lighter colors. (D to F) Localization of the mCherry-tagged (D) Bzp4, (E) Usv101, and (F) Mbs1 with or without *PKA1* deletion was observed. The cells were harvested under basal (nutrient-rich, YPD) conditions and nutrient-starved (YNB without glucose) conditions and stained with Hoechst 33342 to visualize the nucleus. The cells were observed by fluorescence microscopy. Bars, 10 μm.

We next addressed the cellular localization of Bzp4, Usv101, Mbs1, and Hob1 in response to nutrient starvation. To address this issue, we constructed mCherry reporter strains *bzp4*Δ::*BZP4-mCherry*, *usv101*Δ+*USV101-mCherry*, and *mbs1*Δ::*MBS1-mCherry*. Complementation with Bzp4-mCherry and Usv101-mCherry, with mCherry tagging the C terminus, partly restored melanin production in *bzp4*Δ and *usv101*Δ mutants (Fig. 3A and B), suggesting that the Bzp4-mCherry and Usv101-mCherry proteins are partially functional. Complementation with Mbs1-mCherry, with mCherry tagging the C terminus, completely restored melanin production in the *mbs1*Δ mutant (Fig. 3C), suggesting that the Mbs1-mCherry protein is fully functional. In contrast, we attempted to construct a *hob1*Δ::*HOB1-GFP* strain by either N-terminal or C-terminal green fluorescent protein (GFP) tagging, but neither tagged allele was functional (data not shown). We therefore focused on addressing the issue of cellular localization of Bzp4, Usv101, and Mbs1 and their regulation under nutrient-rich and nutrient-starved conditions.

Bzp4 was evenly distributed throughout the cell under the nutrient-rich conditions, but it was rapidly translocated to the nucleus in response to nutrient starvation (Fig. 3D). Similarly, Usv101 also localized to the cytoplasm and nucleus evenly but underwent nuclear translocation in response to nutrient starvation (Fig. 3E). In contrast, Mbs1 was constitutively localized to the nucleus under the nutrient-rich conditions, and its nuclear localization was not changed by nutrient starvation (Fig. 3F). These results indicate that Bzp4 and Usv101 likely undergo nuclear translocation for melanin synthesis through posttranslational regulation in response to nutrient starvation.

Although the cAMP/PKA pathway did not influence expression levels and patterns of *BZP4*, *USV101*, and *MBS1*, the cAMP/PKA pathway may posttranslationally regulate these TFs. To examine this possibility, we deleted *PKA1* in the *bzp4*Δ::*BZP4-mCherry*, *usv101*Δ+*USV101-mCherry*, and *mbs1*Δ::*MBS1-mCherry* strains and monitored cellular localization of Bzp4-mCherry, Usv101-mCherry, and Mbs1-mCherry, respectively. Deletion of *PKA1* did not influence cellular localization of these TFs under nutrient-rich or nutrient-starved conditions (Fig. 3D to F), suggesting that cellular localization of Bzp4, Usv101, and Mbs1 is regulated in a manner independent of the cAMP/PKA pathway.

### Gsk3 and Kic1 kinases function upstream of Hob1 and Bzp4, whereas Pkh202 regulates *MBS1* expression.

**FIG 4:**
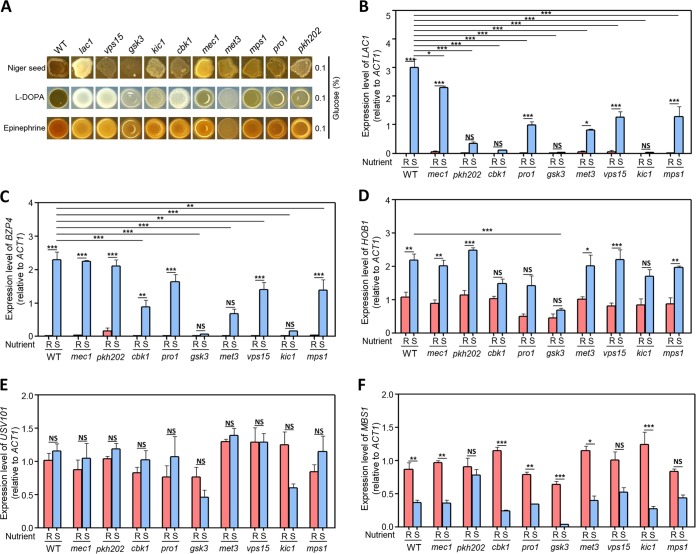
Melanin-regulating kinases in C. neoformans. (A) C. neoformans WT and mutant strains on Niger seed, dopamine, and epinephrine medium. Darker cultures had more effective melanin synthesis than those with lighter colors. (B to F) Quantitative RT-PCR was performed using total RNA of each strain under nutrient-rich (R; YPD) or nutrient-starved (S; YNB without glucose) conditions. The induction of (B) *LAC1*, (C) *BZP4*, (D) *HOB1*, (E) *USV101*, and (F) *MBS1* by nutrient starvation was measured in WT and mutant strains. Three biologically independent experiments were performed with three technical replicates each. Error bars indicate SEM, and statistical differences among gene expression levels were calculated by one-way ANOVA multiple comparisons with Bonferroni’s correction (*, *P < *0.05; **, *P < *0.01; ***, *P < *0.001; NS, not significant).

Because the cAMP/PKA pathway was found to be dispensable for regulation of Bzp4, Usv101, Mbs1, and Usv101, we searched for another potential upstream regulator(s). Previously, we constructed 264 mutant strains representing 129 kinases and found that 49 kinases appeared to be involved in melanin production on Niger seed medium (44). Therefore, we reevaluated the melanin production levels of the 49 kinase mutants on l-DOPA- and epinephrine-containing media as we did for the TF mutant library. In addition to the *pka1*Δ mutant in the cAMP pathway, the following nine kinase mutants exhibited visually evident melanin defects in both media (Fig. 4A): *MEC1*, *PKH202*, *CBK1*, *PRO1*, *GSK3*, *MET3*, *VPS15*, *KIC1*, and *MPS1*. Supporting the observation of defective melanin synthesis, *LAC1* induction mediated by nutrient starvation was significantly reduced in most of these kinase mutants except in the *mec1*Δ mutant (Fig. 4B). In particular, *LAC1* induction was much more strongly reduced in the *pkh202*Δ, *cbk1*Δ, *gsk3*Δ, and *kic1*Δ mutants than in other mutants (Fig. 4B). Notably, Cbk1 and Kic1 are two major components of the regulation of the AceII and morphogenesis (RAM) pathway that is evolutionarily conserved in all eukaryotes and has been implicated in cell cycle regulation, cell separation and polarized growth, mating, maintenance of cell wall integrity, and stress responses (46).

Next, we examined *BZP4* and *HOB1* induction mediated by nutrient starvation in the melanin-defective kinase mutants. *BZP4* induction was most significantly reduced in *gsk3*Δ and *kic1*Δ mutants (Fig. 4C). Notably, *HOB1* induction was abolished in the *gsk3*Δ mutant but not the *kic1*Δ mutant (Fig. 4D). Furthermore, the basal expression levels of *USV101* upon nutrient starvation were also marginally reduced in the *gsk3*Δ and *kic1*Δ mutants (Fig. 4E). Therefore, a possible model is that Gsk3 regulates nutrient starvation-mediated induction of Hob1, which subsequently controls *BZP4* induction under nutrient starvation conditions. However, Kic1 is likely required for *BZP4* induction under nutrient starvation conditions in a Hob1-independent manner. In contrast, Gsk3 and Kic1 appeared to be dispensable for the starvation-mediated repression of *MBS1* (Fig. 4F). Instead, deletion of *PKH202* abolished *MBS1* repression under nutrient depletion conditions (Fig. 4F), indicating that Pkh202 is required for repression of *MBS1*.

**FIG 5:**
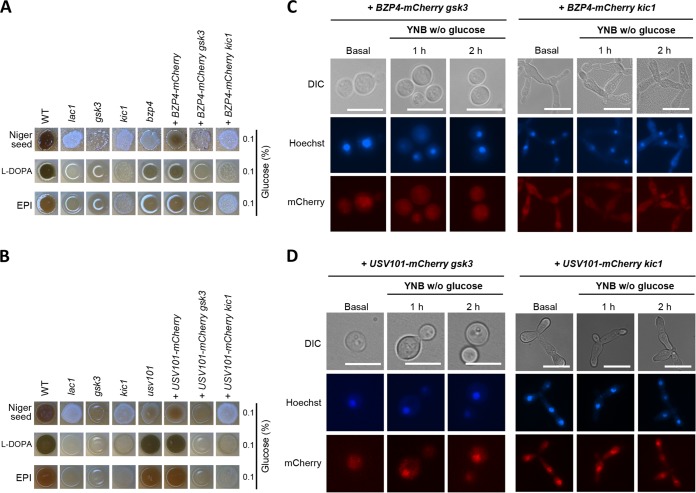
Bzp4-mCherry and Usv101-mCherry constitutively localized in the nucleus. (A and B) C. neoformans WT, *lac1*Δ, *gsk3*Δ, and *kic1*Δ with (A) *bzp4*Δ, *bzp4*Δ::*BZP4-mCherry*, and *bzp4*Δ::*BZP4-mCherry pka1*Δ strains and (B) *usv101*Δ, *usv101*Δ::*USV101-mCherry*, and *usv101*Δ::*USV101-mCherry pka1*Δ mutant strains on Niger seed, dopamine, and epinephrine medium. Darker cultures had more effective melanin synthesis than those with lighter colors. (C and D) Localization of the mCherry-tagged (C) Bzp4 and (D) Usv101 with *GSK3 or KIC1* deletion. The cells were harvested under basal (nutrient-rich, YPD) and nutrient-starved (YNB without glucose) conditions and stained with Hoechst 33342 to visualize the nucleus. The cells were observed by fluorescence microscopy. Bars, 10 μm.

Next, we examined whether Gsk3 and Kic1 can also control nuclear translocation of Bzp4 and Usv101 under nutrient starvation conditions. To this end, we disrupted *GSK3* and *KIC1* in the *bzp4*Δ::*BZP4-mCherry* and *usv101*Δ+*USV101-mCherry* strains and confirmed that each mutant exhibited a melanin-defective phenotype similar to those of the *gsk3*Δ and *kic1*Δ mutants (Fig. 5A and B). As reported before (44), deletion of *KIC1* resulted in aberrant cellular elongation. Surprisingly, deletion of *GSK3* completely abolished nuclear translocation of Bzp4-mCherry but not Usv101-mCherry (Fig. 5C and D). Notably, we found that *KIC1* deletion also abolished the nuclear translocation of Bzp4-mCherry but caused Usv101-mCherry to constitutively localize in the nucleus (Fig. 5C and D). We also examined whether Pkh202 controls constitutive nuclear localization of Mbs1 by disrupting *PKH202* in the *mbs1*Δ::*MBS1-mCherry* strain, but *PKH202* deletion did not affect constitutive nuclear localization of Mbs1 (see Fig. S3 in the supplemental material). Taken together, these data indicate that both Gsk3 and Kic1 positively regulated nuclear translocation of Bzp4, whereas Kic1 suppressed constitutive nuclear localization of Usv101.

10.1128/mBio.02267-19.3 FIG S3 Pkh202 was dispensable for constitutive nuclear localization of Mbs1. (A) C. neoformans WT and mutant strains on Niger seed, dopamine, and epinephrine medium. Darker cultures had more effective melanin synthesis than those with lighter colors. (B) Localization of Mbs1 protein in the *mbs1*Δ::*MBS1*-*mCherry pkh202*Δ mutant strain The cells were harvested under basal (nutrient-rich [YPD]) conditions and nutrient-starved (YNB without glucose) conditions and stained with Hoechst 33342 to visualize the nucleus. The cells were observed by fluorescence microscopy. Bars, 10 μm. Download FIG S3, PDF file, 0.2 MB. Copyright © 2019 Lee et al. 2019 Lee et al. This content is distributed under the terms of the Creative Commons Attribution 4.0 International license.

A question remains about the relationships among Hog1, Cac1, Gsk3, and Kic1. To address this issue, we examined whether *GSK3* and *KIC1* are transcriptionally regulated in the WT strain and the *hog1*Δ and *cac1*Δ mutant strains upon nutrient starvation. Expression of *GSK3* and *KIC1* was induced more than 3-fold in the WT strain upon shifting from YPD to YNB without glucose (see Fig. S4 in the supplemental material). Notably, the WT *GSK3* induction level was equivalent to that of the *hog1*Δ and *cac1*Δ mutants, whereas the *KIC1* induction level was markedly reduced in both mutants. Therefore, HOG and cAMP pathways likely regulate the RAM pathway upstream of Kic1. However, it is still possible that Hog1 and cAMP can interact with Gsk3 in the TOR (Target Of Rapamycin) pathway and/or with Kic1 in the RAM pathway, through posttranslational regulation.

10.1128/mBio.02267-19.4 FIG S4 The role of cAMP/PKA and HOG pathways in regulating expression of *GSK3* and *KIC1*. (A and B) Quantitative RT-PCR was performed using total RNA of each strain under nutrient-rich (R; YPD) or nutrient-starved (S; YNB without glucose) conditions. The induction of (A) *GKS3* and (B) *KIC1* by nutrient starvation was measured in the WT and *hog1*Δ (YSB64) and *cac1*Δ (YSB42) mutant strains. Three biologically independent experiments were performed with three technical replicates each. Error bars indicate standard errors of the means (SEM). Statistical differences among gene expression levels were calculated by one-way ANOVA multiple comparisons performed with Bonferroni’s correction (*, *P < *0.05; **, *P < *0.01; ***, *P < *0.001; NS, not significant). Download FIG S4, PDF file, 0.5 MB. Copyright © 2019 Lee et al. 2019 Lee et al. This content is distributed under the terms of the Creative Commons Attribution 4.0 International license.

### Transcriptome profiles governed by Bzp4, Usv101, Hob1, and Mbs1.

**FIG 6:**
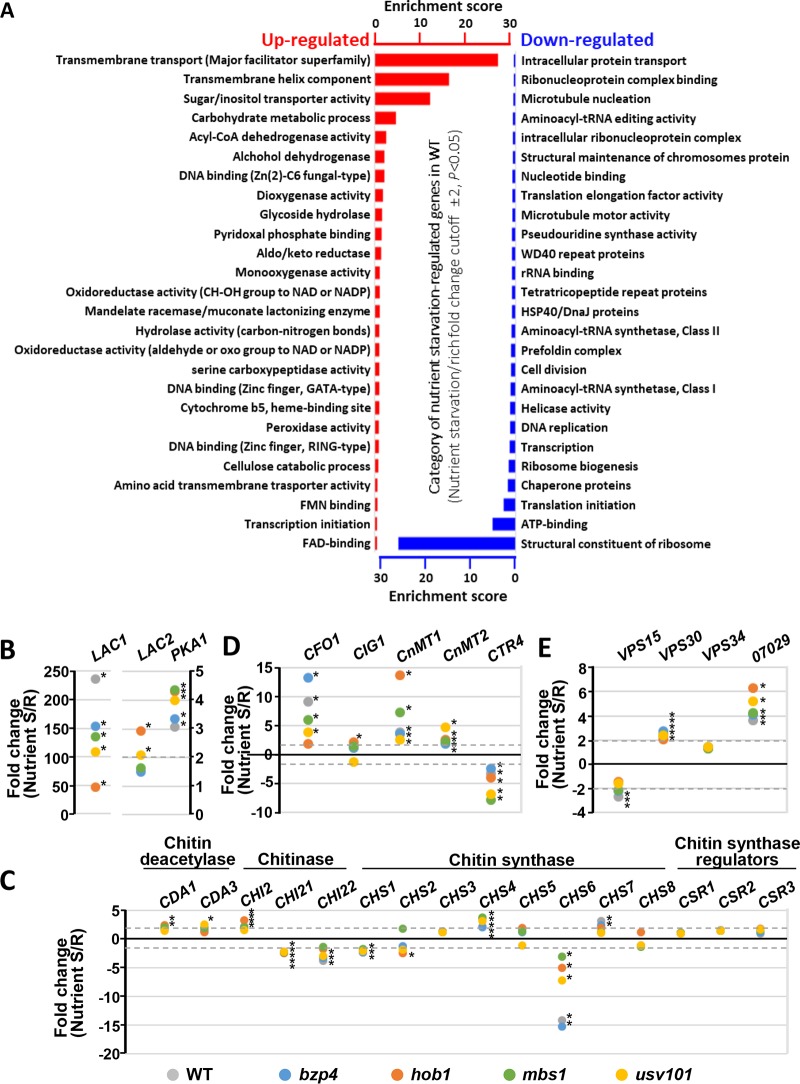
Functional categories of genes regulated in response to nutritional starvation in C. neoformans. (A) DAVID analysis-based enrichment scores of gene ontology (GO) terms for genes upregulated (red) or downregulated (blue) in response to nutritional starvation. The results of DAVID analysis are shown in Data Set S2 in terms of GO term category, and only the enrichment scores for each GO term are shown in the graph. Acyl-CoA, acyl-coenzyme A; FAD, flavin adenine dinucleotide; FMN, flavin mononucleotide. (B to E) Fold changes in expression of genes involved in chitin synthesis and metabolism, iron homeostasis, and vesicle trafficking upon shifting from nutrient-rich to nutrient-starved condition calculated from the RNA-seq data determined for C. neoformans WT, *bzp4*Δ, *hob1*Δ, *mbs1*Δ, and *usv101*Δ strains (Data Set S1). Each asterisk indicates that the fold change in each corresponding strain was more than 2-fold, with statistical significance (*P < *0.05).

Besides induction of the *LAC1* laccase gene, fungal melanization requires a series of biological processes, including extracellular vesicle formation and secretion and anchorage to cell wall components such as chitin (9, 47). To elucidate downstream components and networks governed by the melanin-regulating TFs, we performed RNA sequencing (RNA-seq)-based transcriptome analysis of the *bzp4*Δ, *usv101*Δ, *hob1*Δ, and *mbs1*Δ mutants under nutrient-rich and nutrient-starved conditions. First, we analyzed transcriptome profiles regulated by nutrient starvation in the wild-type strain (Fig. 6A). Under nutrient starvation conditions, genes involved in translation-related processes, protein folding, and DNA replication initiation were significantly downregulated (cutoff of >2-fold change; *P < *0.05) (Fig. 6A) (see Data Set S1 in the supplemental material). In contrast, genes involved in transmembrane transport were highly upregulated (Fig. 6A). These data indicate that C. neoformans reduced its levels of basic biological activities, such as translation and DNA replication, but attempted to obtain more extracellular nutrients under nutrient starvation conditions.

10.1128/mBio.02267-19.9 DATA SET S1 RNA-seq fold change scores of FPKM (*P > *0.05). Download Data Set S1, XLSX file, 0.5 MB. Copyright © 2019 Lee et al. 2019 Lee et al. This content is distributed under the terms of the Creative Commons Attribution 4.0 International license.

10.1128/mBio.02267-19.10 DATA SET S2 Enrichment scores and clustered functional categories of DAVID analysis. Download Data Set S2, XLSX file, 0.1 MB. Copyright © 2019 Lee et al. 2019 Lee et al. This content is distributed under the terms of the Creative Commons Attribution 4.0 International license.

Expression of *LAC1* was more strongly induced than that of *LAC2* or *PKA1* (Fig. 6B). For genes potentially involved in the melanization process, genes encoding products involved in the following categories appeared to be differentially regulated in addition to *LAC1*: copper and iron homeostasis, chitin synthesis, and vesicle trafficking. Among the genes involved in copper and iron homeostasis, expression of ferroxidase gene *CFO1* and metallothionein genes *CnMT1*/*CnMT2* was induced under nutrient starvation conditions (Fig. 6C; see also Data Set S1). In contrast, expression of major copper uptake transporter gene *CTR4* was reduced, with similar expression patterns in *bzp4*Δ, *hob1*Δ, *mbs1*Δ, and *usv101*Δ mutants (Fig. 6D; see also Data Set S1). Interestingly, induction of *CIG1*, which encodes an important mannoprotein required for iron homeostasis (48), was negatively regulated by Hob1. These results indicated that the genes involved in vesicle trafficking and metal homeostasis were modulated by nutrient limitation in C. neoformans. Among the genes involved in chitin synthesis and metabolism, expression of two chitin synthase genes (*CHS4* and *CHS7*) and a chitinase gene (*CHI2*) was significantly upregulated whereas expression of *CHS1*, *CHS6*, *CHI21*, and *CHI22* was downregulated (more than 2-fold change) (Fig. 6D; see also Data Set S1). Previous reports show that chitin synthase Chs3 and one of its regulators, Csr2, are required for retaining melanin pigments in the cell wall, indicating that chitin is important for deposition of melanin precursors (26). Notably, such expression patterns were generally conserved in the *bzp4*Δ, *usv101*Δ, *hob1*Δ, and *mbs1*Δ mutants. However, induction of *CHI2* appeared to be regulated by Usv101, and the levels of induction of *CHS7* or reduction of *CHS6* were reduced in the *hob1*Δ, *mbs1*Δ, and *usv101*Δ mutants (Fig. 6D; see also Data Set S1). In addition, Hob1 and Mbs1 appeared to regulate expression of *CHS1* and *CHI22* (Fig. 6D; see also Data Set S1).

**FIG 7:**
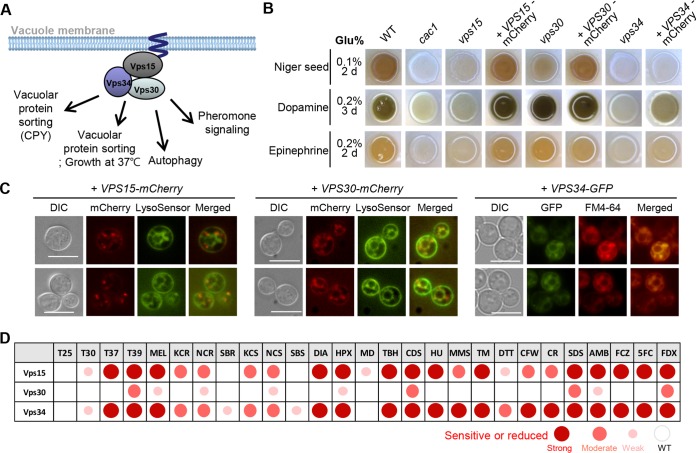
Vesicle trafficking-related genes were involved in melanin production in C. neoformans. (A) Graphic illustration of predicted Vps15/34/30 complex based on the function of S. cerevisiae. CPY, carboxypeptidase Y. (B) C. neoformans WT and mutant strains on Niger seed, dopamine, and epinephrine medium. Darker cultures had more effective melanin synthesis than those with lighter colors. (C) Localization of VPS15-mCherry, VPS30-mCherry, and VPS34-GFP under basal (nutrient-rich, YPD) conditions. LysoSensor or FM4-64 was used as a vacuole-staining dye. The cells were observed by fluorescence microscopy. Bars, 10 μm. (D) Phenotypic heat map of *vps15*Δ, *vps30*Δ, and *vps34*Δ mutants (see data in Fig. S5). T25, T30, T37, and T39, growth rates at 25°C, 30°C, 37°C, and 39°C; MEL, melanin production levels; KCR, YPD plus KCl; NCR, YPD plus NaCl; SBR, YPD plus sorbitol; KCS, YP plus KCl; NCS, YP plus NaCl; SBS, YP plus sorbitol; DIA, diamide; HPX, hydrogen peroxide; MD, menadione; TBH, *tert*-butyl hydroperoxide; CDS, cadmium sulfide; HU, hydroxyurea; MMS, methyl methanesulfonate; TM, tunicamycin; DTT, dithiothreitol; CFW, calcofluor white; CR, Congo red; SDS, sodium dodecyl sulfate; AMB, amphotericin B; FCZ, fluconazole; 5FC, 5-flucytosine; FDX, fludioxonil.

Among the genes involved in vesicle trafficking, expression of *VPS30* (Beclin 1) and CNAG_07029, which is predicted to encode a vesicle-associated membrane protein, was upregulated (Fig. 6E). In contrast, expression of *VPS15*, which is a Ser/Thr kinase involved in vacuolar protein sorting, was downregulated by nutrient starvation. This was unexpected because we previously found that deletion of *VPS15* resulted in severe defects in melanin production (44). Vps30 (also known as Atg6) is homologous to a mammalian autophagy effector, Beclin 1, which is involved in autophagic vesicle nucleation and retromer assembly in association with mammalian class III phosphatidylinositol 3-kinase (PI3K) (a yeast Vps34 ortholog) and p150 (a yeast Vps15 ortholog) (49). To address whether Vps30 and Vps34 participate in melanin production in addition to Vps15, we constructed *vps30*Δ and *vps34*Δ mutants (see Fig. S2 in the supplemental material) and verified the cellular functions of a vacuolar protein sorting-associated complex (Vps34-Vps15-Vps30) to construct the mCherry-tagged complemented strains in each deletion mutant (*vps15*Δ::*VPS15-mCherry*, *vps30*Δ::*VPS30-mCherry*, and *vps34*Δ::*VPS34-GFP*). Strikingly, *VPS34* deletion completely abolished melanin production and *VPS30* deletion reduced melanin production, albeit weakly (Fig. 7B), indicating that the vacuolar protein sorting-associated complex plays an important role in melanin production of C. neoformans. Cellular localization of Vps15, Vps30, and Vps34 was highly correlated to vacuolar membranes in C. neoformans (Fig. 7C). In addition to the critical role in melanin production, Vps15 and Vps34 played pleiotropic roles in stress responses and antifungal drug resistance and their mutants were highly phenotypically similar, but Vps30 had only a minor role in processes outside melanin synthesis (Fig. 7D; see also Fig. S5). These data collectively support the conclusion that Vps15 and Vps30 are major components but that Vps34 is a minor component in a vacuolar protein sorting-associated complex. We also constructed CNAG_07029 deletion mutants (see Fig. S2 in the supplemental material), but melanin production levels were unaffected (see Fig. S6 in the supplemental material). Bzp4, Hob1, Usv101, and Mbs1 were shown not to be involved in modulation of *VPS15*, *VPS30*, *VPS34*, or CNAG_07029 (Fig. 6C), suggesting that another unknown transcription factor(s) might regulate these genes under nutrient starvation conditions in C. neoformans. Taken together, the four TFs may also play some roles in metal homeostasis, chitin synthesis and metabolism, and vesicle trafficking.

10.1128/mBio.02267-19.5 FIG S5 Phenotypic traits of *vps15*Δ, *vps30*Δ, and *vps34*Δ mutants and complemented strains. Spot assays for qualitatively monitoring chemical susceptibility and thermotolerance were performed using the following WT, *vps15*Δ, *vps30*Δ, or *vps34*Δ mutant, and gene-complemented strains: strain *vps15*Δ (YSB1500), strain *vps15*Δ::*VPS15-mCherry* (YSB5509), strain *vps30*Δ (YSB5721), *vps30*Δ::*VPS30* (YSB6191), strain *vps34*Δ (YSB5646), and strain *vps34*Δ::*VPS34* (YSB6555). Each strain was cultured in YPD broth at 30°C overnight, subjected to serial 10-fold dilutions (1 to 10^4^), and spotted on YPD or YP agar containing the indicated concentrations of the chemical agents as indicated in the following panel descriptions. (A) To determine thermotolerance, cells were spotted on YPD medium and further incubated at 25°C, 30°C, 37°C, or 39°C for 2 days and photographed. (B) Osmotic stress was induced with NaCl, KCl, or sorbitol. (C) Heavy metal stress was induced with CdSO_4_ (cadmium sulfate). (D) Oxidative stress was induced with the following agents: diamide, H_2_O_2_ (hydrogen peroxide), MD (menadione), and tBOOH (*tert*-butyl hydroperoxide). (E) DNA damage stress was induced with the following DNA-damaging agents: HU (hydroxyurea) and MMS (methyl methanesulfonate). (F) Endoplasmic reticulum (ER) stress was induced with the following ER stress agents: TM (tunicamycin) and DTT (dithiothreitol). (G) The following fungal cell wall/membrane-disturbing agents were used: CFW (calcofluor white), CR (Congo red), and SDS (sodium dodecyl sulfate). (H) The following antifungal agents were used: FCZ (fluconazole), AMB (amphotericin B), 5-FC (5-flucytosine), and FDX (fludioxonil). Each plate was incubated at 30°C and photographed after 2 to 5 days. Download FIG S5, PDF file, 0.9 MB. Copyright © 2019 Lee et al. 2019 Lee et al. This content is distributed under the terms of the Creative Commons Attribution 4.0 International license.

10.1128/mBio.02267-19.6 FIG S6 CNAG_07029 did not affect the melanin production. C. neoformans WT and CNAG_07029 mutant strains (Table S1) were spotted on medium plates containing Niger seed, dopamine, and epinephrine. Darker cultures had more effective melanin synthesis than those with lighter colors. Download FIG S6, PDF file, 0.7 MB. Copyright © 2019 Lee et al. 2019 Lee et al. This content is distributed under the terms of the Creative Commons Attribution 4.0 International license.

**FIG 8:**
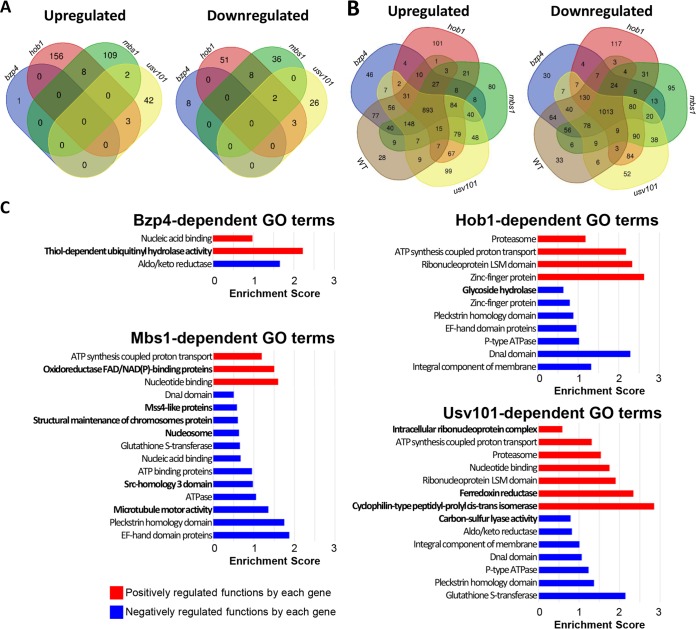
Transcriptome profiles governed by Bzp4, Usv101, Hob1, and Mbs1 under nutrient-rich and nutrient-starved conditions. (A and B) The number of genes whose expression was significantly upregulated or downregulated at least 2-fold in the *bzp4*Δ, *hob1*Δ, *mbs1*Δ, and *usv101*Δ mutants compared with the WT strain under nutrient-rich conditions (YPD) (A) or nutrient-starved conditions (YNB without glucose) (B) are indicated in Venn diagrams. (C) Enrichment scores of DAVID analysis of each functional category in the indicated deletion mutants. Red and blue bars indicate upregulated and downregulated categories, respectively. The GO results of DAVID analysis are reshown in Data Set S2, and only the enrichment scores for each GO term are shown in the graph. Bold letters indicate GO terms corresponding to the indicated mutant strain but not to the other mutant strains.

We next comprehensively analyzed the downstream networks of Bzp4, Usv101, Hob1, and Mbs1 by comparing the transcriptome profiles of *bzp4*Δ, *usv101*Δ, *hob1*Δ, and *mbs1*Δ mutants under nutrient-rich and nutrient-starved conditions. Under the basal conditions, *BZP4* deletion affected only a small number of genes (total, 9 genes; 2-fold cutoff) (Fig. 8A; see also Data Set S1), which is not surprising because Bzp4 is mainly localized in the cytoplasm under nutrient-rich conditions. Similarly, most genes differentially regulated in *usv101*Δ, *hob1*Δ, and *mbs1*Δ mutants under the nutrient-rich conditions could not be significantly classified into functional categories by GO term analysis. Under nutrient starvation conditions, however, many genes were upregulated or downregulated by Bzp4, Usv101, Hob1, and Mbs1 (Fig. 8B), and we grouped these into functional categories (Fig. 8C). Although the TFs shared some functions, with this exception, each TF appeared to regulate unique biological functions: Bzp4 regulated hydrolase activities; Usv101 regulated peptidyl-prolyl *cis*-*trans* isomerases, ferredoxin reductases, ribonucleoproteins, and carbon-sulfur lyases; Hob1 regulated glycoside hydrolases; and Mbs1 regulated oxidoreductases, microtubule motors, nucleosomes, chromosome, and Mss4-like and Src-homology 3 domain proteins. Collectively, Bzp4, Usv101, Hob1, and Mbs1 have redundant and distinct roles in a number of other biological processes as well as in melanin production in C. neoformans.

### The Sks1 kinase coregulated by Usv101, Hob1, and Mbs1 is involved in melanin production.

**FIG 9:**
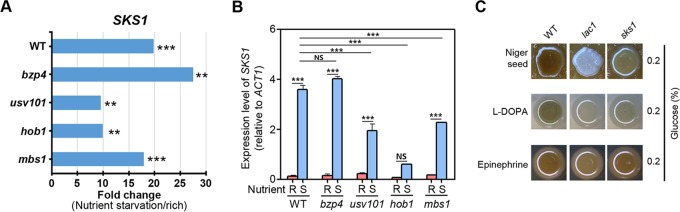
The role of the Sks1 kinase downstream of Usv101, Hob1, and Mbs1 in melanin production. (A) Fold change in *SKS1* expression upon shifting from nutrient-rich conditions (YPD) to nutrient-starved conditions (YNB without glucose) from the RNA-seq data of the C. neoformans WT and indicated mutant strains (Data Set S1). Double and triple asterisks indicate *P* values of <0.01 and <0.001, respectively. NS, not significant. (B) The expression level of *SKS1* was measured in WT and mutant strains under nutrient-rich (R; YPD) or nutrient-starved (S; YNB without glucose) conditions. Three biologically independent experiments were performed with three technical replicates each. Error bars indicate SEM, and statistical differences among gene expression levels were calculated by one-way ANOVA multiple comparisons with Bonferroni’s correction (*, *P < *0.05; **, *P < *0.01; ***, *P < *0.001; NS, not significant). (C) C. neoformans WT and mutant strains on Niger seed, dopamine, and epinephrine medium. Darker cultures had more effective melanin synthesis than those with lighter colors.

The *SKS1* gene, which encodes a putative Ser/Thr protein kinase(s) and is known to be involved in adaptation to low concentrations of glucose in Saccharomyces cerevisiae (50), was upregulated upon nutrient starvation and coregulated by the core TFs in C. neoformans. On the basis of the RNA-seq data, *SKS1* expression was induced by Usv101, Hob1, and Mbs1 but was weakly repressed by Bzp4 (Fig. 9A). To augment these data, we performed quantitative reverse transcription-PCR (qRT-PCR) analysis and found that *SKS1* expression was indeed highly upregulated in response to nutrient starvation in the wild-type strain (Fig. 9B). Induction of *SKS1* expression was significantly reduced in the *usv101*Δ, *hob1*Δ, and *mbs1*Δ mutants and was marginally but not significantly reduced in the *bzp4*Δ mutant (Fig. 9B). The *sks1*Δ mutant was defective in melanin production in Niger seed and l-DOPA media (Fig. 9C), indicating that Sks1 is one of melanin-regulating signaling components downstream of Usv101, Hob1, and Mbs1.

## DISCUSSION

**FIG 10:**
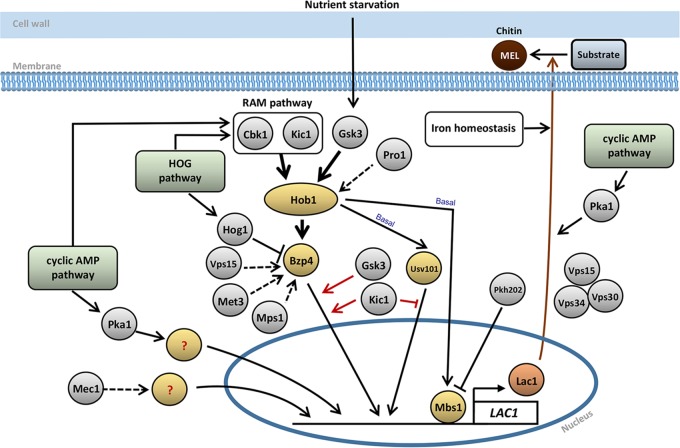
The proposed regulatory mechanism of melanin-regulating signaling pathways in C. neoformans. In response to nutrient (particularly glucose) starvation, expression of the *LAC1* laccase gene, which encodes a rate-limiting enzyme for eumelanin production in C. neoformans, is induced in manners dependent on Bzp4, Usv101, and Mbs1. Hob1 regulates nutrient starvation-mediated induction of *BZP4* and basal expression of *USV101* and *MBS1*. The RAM pathway, consisting of Cbk1 and Kic1 kinases, and Gsk3 mainly control induction of *HOB1*, whereas the Pro1 kinase weakly regulates it. Pkh202 suppresses nutrient starvation-mediated repression of *MBS1*. Bzp4 and Usv101 undergo nuclear translation upon nutrient starvation, but Mbs1 is constitutively localized in the nucleus. Both Gsk3 and Kic1 kinases regulate nuclear translocation of Bzp4, and Kic1 represses nuclear translocation of Usv101. *BZP4* expression is also weakly regulated by Vps15, Met3, and Mps1 kinases. The HOG pathway negatively regulates *BZP4* induction. However, the cAMP/PKA pathway and the Mec1 kinase promote *LAC1* induction in a manner independent of Bzp4, Usv101, Hob1, and Mbs1 TFs. Both HOG and cAMP pathways promote induction of *KIC1* in the RAM pathway upon nutrient starvation. The Vps15-Vps30-Vps34 complex is likely to be involved in melanin (MEL) production through vesicle trafficking. The cAMP pathway is also involved in laccase localization.

In this study, we elucidated complex signaling networks regulating the production of melanin, an antioxidant polyphenol pigment that serves as a key virulence factor for C. neoformans (summarized in Fig. 10). Here, we found that four core TFs, Hob1, Usv101, Bzp4, and Mbs1, played a pivotal role in induction of *LAC1* under nutrient starvation conditions. Hob1 functioned upstream of Bzp4 and Usv101, governing nutrient starvation-mediated *BZP4* induction and basal expression levels of Usv101. In contrast, Mbs1 was independently regulated as both a repressor and activator for melanin production. Gsk3 and Kic1 kinases functioned upstream of Hob1 and Bzp4 whereas Pkh202 regulated Mbs1 suppression in response to nutrient starvation. Both Gsk3 and Kic1 governed the nuclear translocation of Bzp4 from the cytoplasm during melanin synthesis, whereas Kic1 suppressed constitutive nuclear translocation of Usv101. The four melanin-regulating core TFs regulated distinct and redundant sets of downstream effector genes under nutrient starvation conditions, including additional signaling components such as the Sks1 protein kinase. Therefore, our study clearly demonstrated that melanin-regulating signaling networks are far more complicated than originally expected based on the dominant role of the cAMP/PKA and HOG pathways in melanin synthesis in C. neoformans.

Among the four melanin-regulating core TFs, some functions and regulatory mechanisms have been partially characterized in C. neoformans (43, 51, 52). It was previously shown that Usv101 is not only required for production of melanin but is also involved in capsular shedding; although deletion of *USV101* renders cryptococcal cells hypercapsular, the *usv101*Δ mutant is significantly defective in early survival within a host (43, 51). Gish et al. also monitored the transcriptome profiles of the *usv101*Δ mutant and proposed that the melanin defects observed could have been due to reduced expression of *CTR1*, encoding a high-affinity copper transporter, because copper is required for the catalytic function of laccase (51). Our data suggest that *USV101* also directly regulates expression of *LAC1*. Furthermore, they also reported that expression of three genes (*AGS1*, *CHS5*, and *SKN1*) involved in cell wall polysaccharides is significantly reduced in the *usv101*Δ mutant (51). Because cell wall integrity is required for melanin deposition, cell wall irregularities could contribute to the melanin defects observed in the *usv101*Δ mutant. Our independent transcriptome analysis supports this finding; we found that expression of *CHI2*, *CHS5*, and *CHS7* was induced (approximately 2-fold) by nutrient starvation in the wild-type strain but not in the *usv101*Δ strain.

Our previous systematic analysis of C. neoformans TFs demonstrated that Bzp4 plays positive roles in both melanin and capsule biosynthesis but is not required for virulence (43). Interestingly, a recent genome-wide association study of a genetically diverse population of C. neoformans demonstrated that a lack of melanization is closely associated with loss-of-function mutations in *BZP4* (53), further indicating that Bzp4 is responsible for different melanization levels among diverse clinical and environmental isolates. Mbs1 is an APSES (ASM-1, Phd1, StuA, EFG1, and Sok2)-like TF involved in flucytosine susceptibility in a Tco2/Hog1-dependent manner (52), with *mbs1*Δ mutants exhibiting increased susceptibility. In addition to its role in melanin synthesis, Mbs1 is involved in ergosterol biosynthesis, stress responses, and titan cell formation. *MBS1* deletion weakly attenuates the virulence of C. neoformans (52).

Our prior studies showed that the homeobox TF Hob1, which is a key regulator of Bzp4 and Usv101, plays pleiotropic roles in resistance to environmental stress and is required for the pathogenicity of C. neoformans (43). More recently, we discovered that Hob1 is not only required for the survival of C. neoformans in the lungs but is also critical for crossing the blood-brain barrier and survival in the brain parenchyma (K.-T. Lee, J. Hong, D.-G. Lee, M. Lee, S. Cha, Y.-G. Lim, K.-W. Jung, A. Hwangbo, Y. Lee, S.-J. Yu, Y.-L. Chen, J.-S. Lee, E. Cheong, Y.-S. Bahn, submitted for publication). Most notably, Hob1 controls induction of several brain infection-related genes, such as inositol transporter genes (*ITR1a* and *ITR3c*) and a metalloprotease gene (*MPR1*), and other virulence-regulating core TFs, such as *PDR802* and *SRE1*, under host-mimic conditions. Nevertheless, in Cryptococcus gattii, Hob1 does not play evident roles in growth, stress responses, and melanin production, suggesting that C. gattii may have different regulatory networks for melanin production.

Our study provided insights into the interconnectivity of known and newly identified pathways of melanin production. Previous work demonstrated that the HOG pathway negatively regulates melanin production and that its inhibition completely restores normal melanin production in the cAMP/PKA pathway mutants of C. neoformans (41, 42), and, here, we described how deletion of *HOG1* increased *BZP4* induction, which may result in increased expression of *LAC1* and melanin production. However, on the basis of the data in this study, the cAMP/PKA pathway, previously described as the critical signaling pathway mediating melanin production by controlling *LAC1* induction (54) and laccase localization (55), appeared to be dispensable for regulation of the four TFs. Deletion of *PKA1* did not affect the induction of *BZP4* and *HOB1* expression or nuclear translocation of *BZP4* and *USV101* and did not reduce expression and constitutive nuclear localization of *MBS1* under nutrient starvation conditions. Nevertheless, it is still possible that the cAMP/PKA pathway may regulate transcriptional coactivators that are structurally and/or functionally associated with the four TFs.

Among the upstream regulators of the four melanin-regulating core TFs, the Gsk3-dependent signaling pathway appears to be the most important. We found that Gsk3 was required for full induction of *LAC1*, induction and nuclear translocation of *BZP4*, induction of *HOB1*, and expression of *USV101* and *MBS1* under nutrient starvation conditions. Gsk3 is homologous to the glycogen synthase kinase 3 (GSK3) family members that are evolutionarily conserved in all eukaryotes (56). In mammals, the function of Gsk3 is associated with the PI3K/AKT/mTOR signaling network for regulation of growth, proliferation, and metabolism (56). In S. cerevisiae, Rim11, a Gsk3 homolog, is required for phosphorylation and for promoting the formation of the Ime1 and Ume6 complex, which promotes early meiosis gene expression for sporulation (57). Notably, the cAMP/PKA pathway negatively regulates the function of Rim11, and the kinase activity of Rim11 is thereby inhibited by the presence of nutrients in a cAMP/PKA-dependent manner (57). Therefore, it is conceivable that nutrient depletion for melanin production may activate Gsk3 activity, which subsequently mediates the nuclear translocation of Bzp4 and induction of *LAC1*. However, because deletion of *PKA* did not affect the nuclear translocation of Bzp4 or *LAC1* induction and because both Gsk3 and cAMP/PKA pathways positively regulate melanin production, it is not likely that Gsk3 is regulated by the cAMP/PKA pathway. Instead, as Chang et al. previously reported, Gsk3 is involved in the sterol regulatory element-binding protein (SREBP) pathway and required for the survival of C. deneoformans (B-3501 strain) under low-oxygen conditions (58). Similarly, we previously reported that *gsk3*Δ mutants constructed in the H99 strain background exhibit increased susceptibility to fluconazole and SDS, a membrane destabilizer, strongly suggesting that Gsk3 is involved in the SREBP pathway of C. neoformans (43). However, given that deletion of *SRE1*, which encodes a key TF in the SREBP pathway, does not alter melanin production levels (43), we speculate that Gsk3 may control Bzp4 and melanin production in a SREBP-independent manner.

Here, we demonstrated that the RAM pathway is a major melanin-regulating pathway that governs the induction and nuclear translocation of Bzp4 and, thereby, laccase gene expression. This pathway has been best characterized in S. cerevisiae, in which the RAM pathway consists of two Ser/Thr protein kinases, Kic1 and Cbk1, and their associated proteins. The Kic1 protein kinase, associated with Hym1 and Sog2, phosphorylates and regulates Cbk1, which binds to and is regulated by Mob2 (46). The activated Cbk1 controls cell separation, polarized mRNA localization, secretion, and stress signaling through regulation of AceII, Ssd1, Sec2, and Bck2, respectively. Deletion of the RAM components led to constitutive hyperpolarization in C. neoformans (59), instead of the loss of polarity seen in S. cerevisiae. Interestingly, our data showed that deletion of *KIC1* reduced *BZP4* induction more than *CBK1* induction, suggesting that Kic1 may have another downstream target protein(s) for regulation of the Bzp4 TF.

We also described here that Pkh202 could be the potential upstream kinase that regulates nutrient starvation-mediated *MBS1* repression. It is therefore conceivable that the reduced melanin production observed in the *pkh202*Δ mutant could have been due to its inability to repress *MBS1* expression under nutrient starvation conditions. Pkh202 is orthologous to human phosphoinositide-dependent kinase 1 (hPDK1); S. cerevisiae contains three hPDK1 orthologs, Pkh1, Pkh2, and Pkh3, which play roles in cell wall integrity, sphingolipid biosynthesis, endocytosis, eisosome formation, flippase activity, and RNA metabolism (60–63). Two hPDK1 orthologs, Pkh201 and Pkh202, have been described in C. neoformans, and Pkh202, but not Pkh201, plays a number of critical roles in growth, stress responses, antifungal drug susceptibility, and pathogenicity of C. neoformans (44, 64–66). Although Pkh202 is required for activation of the cell wall integrity pathway by regulating phosphorylation of the Mpk1 mitogen-activated protein kinase (MAPK) (66), given that the *mpk1*Δ mutant was not defective in melanin production in our study, the Mpk1 MAPK pathway is not likely to be involved in Pkh202-dependent Mbs1 regulation.

Analysis of the transcriptomes of *usv101*Δ, *bzp4*Δ, *hob1*Δ, and *mbs1*Δ mutants demonstrated that the melanin-regulating core TFs have redundant and distinct sets of downstream genes in addition to their common effector gene, *LAC1*. This redundancy is not surprising because these TFs are involved in a number of other phenotypic traits of C. neoformans (43). Given the cytoplasmic location and narrow role of *BZP4* governing melanin and capsule production, it is not surprising that *BZP4* deletion regulated only 9 genes (1 upregulated and 8 downregulated) under basal conditions. Usv101 also has limited roles (capsule production, membrane integrity, and melanin production) in C. neoformans, and the deletion of *USV101* correspondingly affected a moderate number of genes (47 upregulated and 31 downregulated). In contrast, reflecting the pleiotropic role of Hob1 in growth, differentiation, and stress responses, *HOB1* deletion affected (more than 2-fold) the upregulation and downregulation of 167 and 64 genes, respectively, even under nutrient-rich conditions. Similarly, deletion of *MBS1*, which also has pleiotropic roles in differentiation, stress responses, and antifungal drug susceptibility, altered expression of 165 genes under the basal conditions. Because Mbs1 is constitutively localized into the nucleus, its deletion was likely to upregulate a large number of genes (119 genes).

Overall, each TF regulated more genes under nutrient-starved conditions than under nutrient-rich conditions. In wild-type C. neoformans, 1,361 genes were upregulated in response to nutrient starvation and 1,485 genes were downregulated. Most of these genes (893 upregulated and 1,013 downregulated genes) were not transcriptionally affected by deletion of *USV101*, *BZP4*, *HOB1*, or *MBS1*, suggesting that other TFs were involved in adaptation to nutrient starvation, as expected. Potential TFs required for adaptation to nutrient starvation include *HXL1* in the unfolded protein response pathway and two putative essential TFs (CNAG_00883 and CNAG_04798) whose expression was also upregulated in our RNA-seq analysis. Notably, expression of *BUD32* (tRNA modification), *MET3* (methionine metabolism), and *MPS1* (cell cycle regulation) and of 18 putative essential kinases was repressed under nutrient starvation conditions, further supporting the finding that basic cellular functions, including translation and transcription, were downregulated during nutrient starvation. Although we identified many potential downstream target genes of the melanin-regulating core TFs, a number of them were not functionally characterized or annotated.

## MATERIALS AND METHODS

### Strains of C. neoformans and melanin induction conditions.

Cryptococcus neoformans strains used in this study are listed in Table S1 in the supplemental material. Strains were cultured and maintained in yeast extract-peptone-dextrose (YPD) medium. For the melanin production assay, strains were inoculated into 2 ml of YPD broth and cultured overnight at 30°C in a shaking incubator. Cells were spun down, washed twice with phosphate-buffered saline (PBS), and resuspended in 1 ml PBS. Each strain was spotted (3 μl) on Niger seed medium (70 g Niger seed and 20 g Bacto agar per liter), dopamine medium, or epinephrine agar medium (1 g l-asparagine, 3 g KH_2_PO_4_, 250 mg MgSO_4_, 1 mg thiamine, 5 μg biotin, and 100 mg l-DOPA or epinephrine hydrochloride per liter) with a limited glucose concentration (0.1% or 0.2%). Cells were incubated at 30°C and photographed for 1 to 3 days daily under a microscope (SMZ-168; Motic) at ×10 magnification.

### Gene disruption and complementation.

The gene deletion mutants used in this study are listed in Table S1. Each gene disruption cassette contained nourseothricin, G418, or hygromycin B selection marker (*NAT*, *NEO*, or *HYG*, respectively) and was amplified by double-joint PCR (DJ-PCR) with the screening primers listed in Table S2 as previously reported (44). Target gene deletion was confirmed by Southern blotting (see Fig. S2 in the supplemental material). To verify the phenotypes observed in *bzp4*Δ, *mbs1*Δ, *usv101*Δ, *vps15*Δ, *vps30*Δ, and *vps34*Δ mutants in C. neoformans strain H99, tagged, complemented strains were constructed. The promoter and open reading frame were amplified with specific primer pairs LP/RP listed in Table S2 (XhoI/NotI ends for *BZP4*; XbaI/NotI ends for *USV101*; NotI ends for *MBS1*, *VPS30*, and *VPS34*) and cloned into pTOP-V2 vector to confirm the sequence. Due to the long length of *VPS15*, 5′ and 3′ fragments were PCR amplified with LP1/RP1 (NheI/ApaI ends) and LP2/RP2 (NheI/ApaI ends) primer pairs, respectively, and separately cloned into pTOP-V2 vector. The 5′ fragment was subcloned into the 3′ fragment in pTOP-V2 and sequenced. Each insertion in pTOP plasmids was subcloned into pNEO-mCherry or pNEO-GFP vectors, linearized with a specific enzyme (AflII for pNEO-Bzp4-mCherry, EcoRV for pNEO-USV101-mCherry, SacII for pNEO-Mbs1-mCherry, AsiSI for pNEO-VPS15-mCherry, and XhoI for pNEO-VPS30-mCherry and pNEO-VPS34-GFP), and introduced by biolistic transformation into corresponding mutant strains (Table S1). The targeted or ectopic integration of each gene was confirmed by diagnostic PCR (Table S2).

### Cellular localization and vacuole-staining assays.

For the Bzp4 and Mbs1 localization study, *BZP4*-*mCherry*, *BZP4*-*mCherry pka1*Δ, *BZP4*-*mCherry kic1*Δ, *BZP4*-*mCherry gsk3*Δ, *MBS1*-*mCherry*, and *MBS1*-*mCherry pka1*Δ strains (Table S2) were cultured overnight in 50 ml of YPD broth at 30°C in a shaking incubator. All strains were subcultured in 40 ml fresh YPD broth until the optical density at 600 nm (OD_600_) reached 0.6 to 0.8, at which point they were washed with PBS, resuspended in 40 ml of YNB liquid medium without glucose, and further incubated at 30°C in a shaking incubator. One milliliter of each sample was fixed at each time point (0, 60, and 120 min) using 10% paraformaldehyde. Fixed cells were stained with Hoechst 33342 to visualize the nucleus and observed through a differential interference contrast (DIC) fluorescence microscope (BX51; Olympus).

To covisualize the vacuoles and cellular location of Vps15, Vps30, and Vps34, FM4-64 (Invitrogen) or LysoSensor green (Thermo Fisher) was used as a vacuole-staining dye. For *vps15*Δ::*VPS15-mCherry* (YSB5509) and *vps30*Δ::*VPS30–mCherry* (YSB6191), each strain was cultured overnight in YPD broth and subcultured until the OD_600_ reached 0.8. One milliliter of cells was spun down and mixed with fresh liquid YPD medium supplemented with 1 μl of 1 mM LysoSensor green. Cells were further incubated at 30°C for 30 min. For *vps34*Δ::*VPS34–GFP* (YSB6555), the strain was cultured overnight in YPD broth and subcultured to an OD_600_ of 0.8. A 1-ml volume of cells was spun down and resuspended with 5 μg/ml FM4-64 dye in ice-cold Hanks’ balanced salt solution (HBSS; Gibco) and kept on ice for 30 min. The cells were pelleted by centrifugation, washed three times with HBSS, and resuspended with 100 μl of HBSS. On a glass slide, 5 μl of the cells and 5 μl of mounting solution (Biomeda) were mixed, covered with a cover glass, and observed by DIC fluorescence microscopy (BX51; Olympus).

### Total RNA preparation and quantitative RT-PCR.

WT and mutant strains were inoculated into 50 ml of YPD broth and cultured overnight at 30°C in a shaking incubator. Cells were subcultured in 80 ml fresh YPD broth until the OD_600_ reached 0.6 to 0.8. A 40-ml volume of the culture was placed in a liquid nitrogen tank as a basal control sample, and the remaining 40-ml volume was spun down, washed three times with PBS, and resuspended in 40 ml of YNB medium without glucose. After resuspension, cells were further incubated at 30°C in a shaking incubator for 2 h. Incubated cells were spun down, frozen in liquid nitrogen, and lyophilized overnight. Total RNA was isolated by TRIzol extraction (easy-BLUE, iNtRON). cDNA was synthesized by the use of reverse transcriptase (Thermo Scientific). The levels of expression of all genes (*LAC1*, *ACT1*, *BZP4*, *USV101*, *HOB1*, and *MBS1*) were analyzed by quantitative real-time PCR (CFX96 real-time system; Bio-Rad) using specific primer pairs (Table S2) and *ACT1* expression as a normalization control.

### RNA-seq and data analysis.

Total RNAs prepared as described above were purified with a commercial kit (RNeasy minikit; Qiagen). The concentration was measured with RNA detection dye (Quant-IT RiboGreen; Invitrogen). The quality of the RNA was verified by the use of TapeStation RNA ScreenTape (Agilent). RNA samples with an RNA integrity number (RIN) greater than 7.0 were used to construct the cDNA library (TruSeq mRNA sample prep kit; Illumina) according to manufacturer protocol. We processed the data from the sequencer (HiSeq 2500; Illumina) by using Illumina Casava1.7 software for base calling. The sequenced reads were trimmed to remove the adaptor sequence and masked for low-complexity or low-quality sequence by using Trimmomatic v0.32 with TruSeq3-PE.fa and MINLEN:36. The reads were aligned to the C. neoformans H99 genome from FungiDB using Tophat v2.0.13 (67) with the Bowtie v2.2.3 algorithm (68). Tophat was used with the “-G” option and other parameters set to default. Transcript assembly and abundance estimations were performed using Cufflinks v2.2.1 (67). To correct the sequence expression count bias, we used the “–max-bundle-frags 50000000” option. The isoform transcripts were also calculated, and the relative transcript abundances for each gene were measured as the sum of the numbers of fragments in the exon model quantified as fragments per kilobase per million (FPKM) using Cufflinks. We performed statistical analysis to identify differentially expressed genes (DEG). To facilitate log2 transformation, a value of 1 was added to each FPKM value representing filtered genes. Filtered data were subjected to log2 transformation and to quantile normalization. The statistical significance of the differential expression data was determined using independent *t* tests and fold change analyses in which the null hypothesis was that no difference existed between groups. The false-discovery rate (FDR) was controlled by adjusting *P* values using the Benjamini-Hochberg algorithm. For DEG sets, hierarchical clustering analysis was performed using complete linkage and Euclidean distance as a measure of similarity. Functional annotation analysis was performed for DEG by the use of DAVID (http://david.abcc.ncifcrf.gov/) to understand the biological functions in the large list of genes. We selected DAVID-defined defaults, including keyword-based functional categories (GO term “BP direct,” GO term “CC direct,” GO term “MF direct,” and protein domain databases), and the modified Fisher exact *P* value (EASE score) was 0.1. The clustered GO group is listed in Data Set S2 in the supplemental material. The overall score for the group determined on the basis of the EASE scores of all term members was represented as an enrichment score.

### Data availability.

Our RNA-seq data were deposited in the Gene Expression Omnibus (GEO) database (accession number GSE131891).

10.1128/mBio.02267-19.7 TABLE S1 C. neoformans strains used in this study. Download Table S1, DOCX file, 0.03 MB. Copyright © 2019 Lee et al. 2019 Lee et al. This content is distributed under the terms of the Creative Commons Attribution 4.0 International license.

10.1128/mBio.02267-19.8 TABLE S2 Primers used in this study. Download Table S2, DOCX file, 0.02 MB. Copyright © 2019 Lee et al. 2019 Lee et al. This content is distributed under the terms of the Creative Commons Attribution 4.0 International license.
