# Supplementary figures and images for: Unraveling Melanin Biosynthesis and Signaling Networks in Cryptococcus neoformans
Source: mBio. 2019 Oct 1;10(5):e02267-19. doi: 10.1128/mBio.02267-19 (PMC6775464; doi:10.1128/mBio.02267-19)

Figure S1 (Lee et al.)

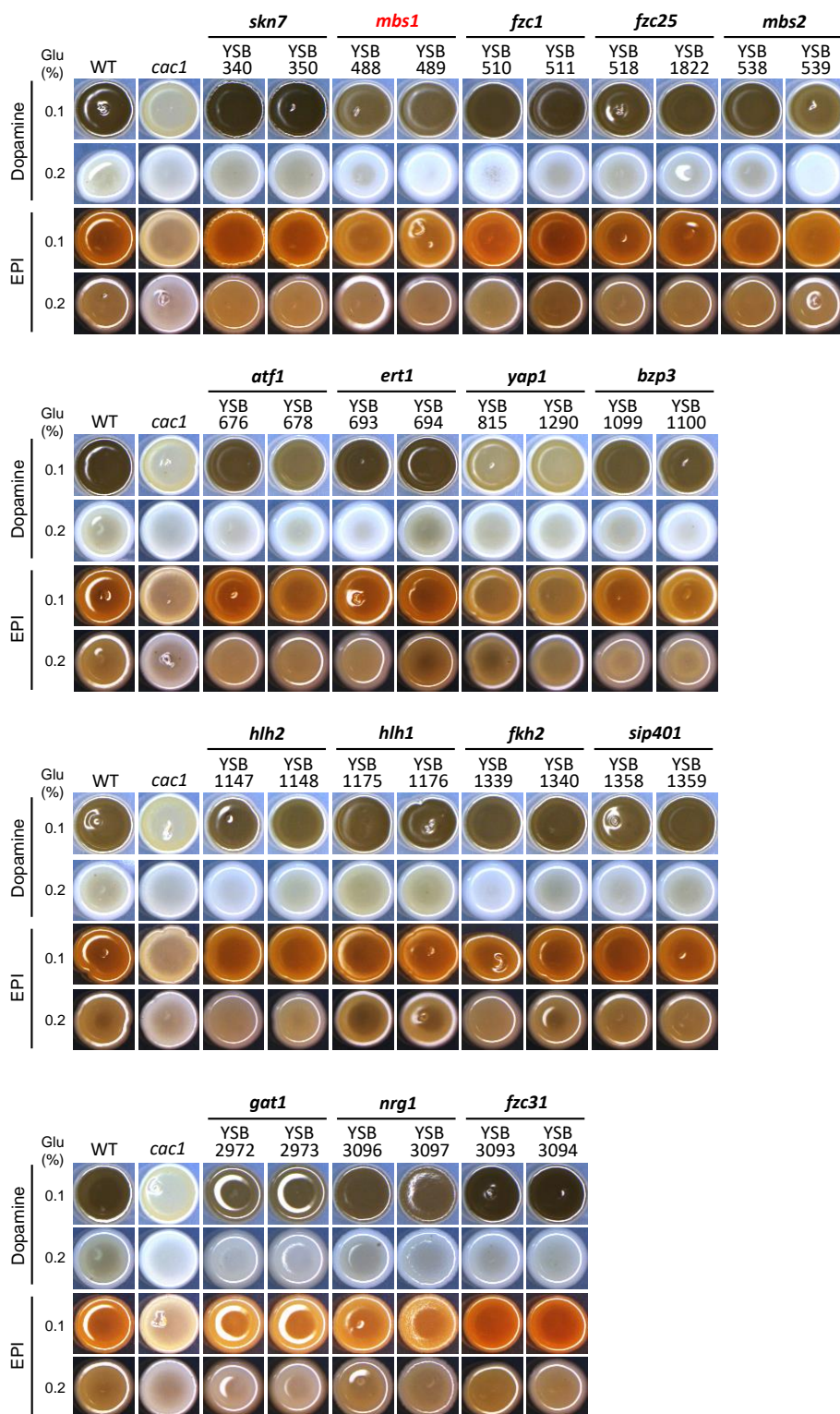

Continued

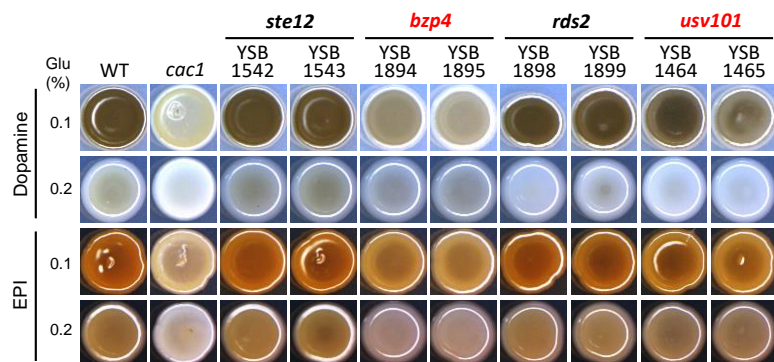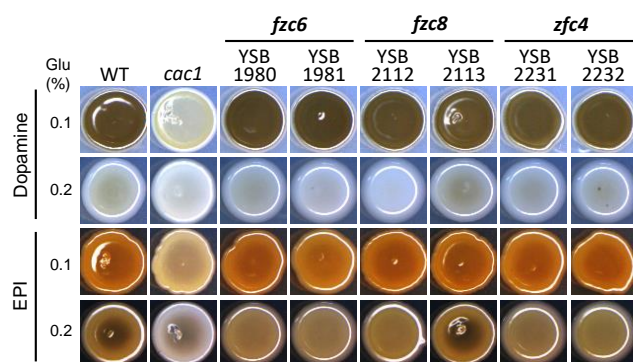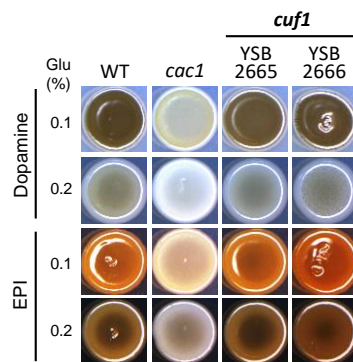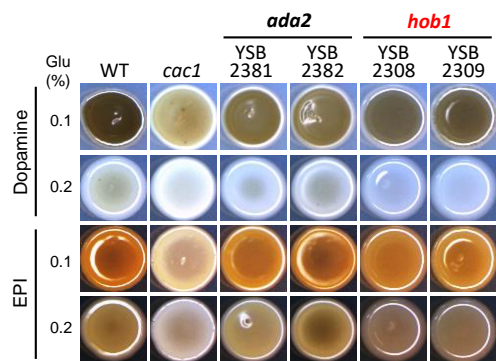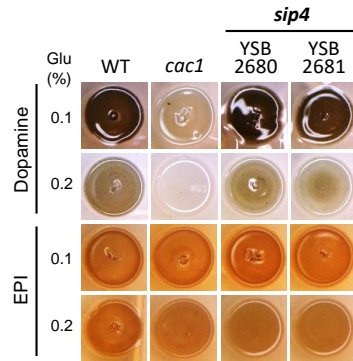

Supplement: FIG S1 [file mBio.02267-19-sf001.pdf]

**Figure S3 (Lee et al.)**

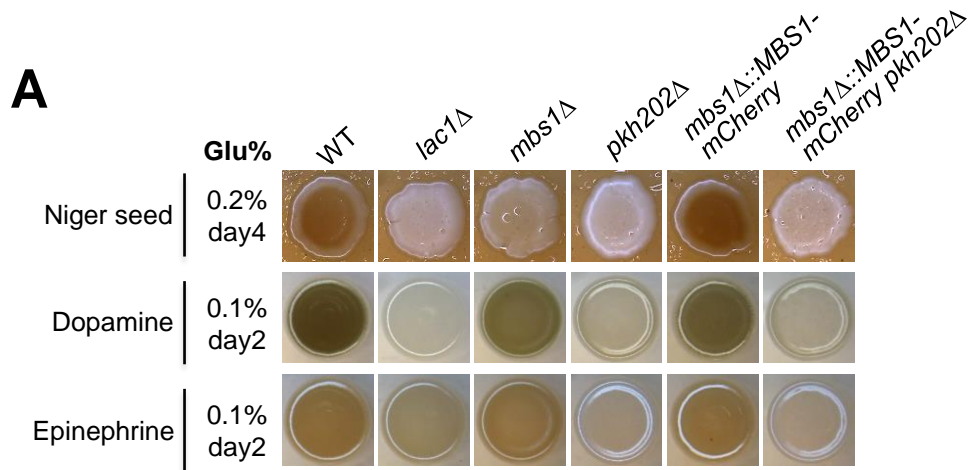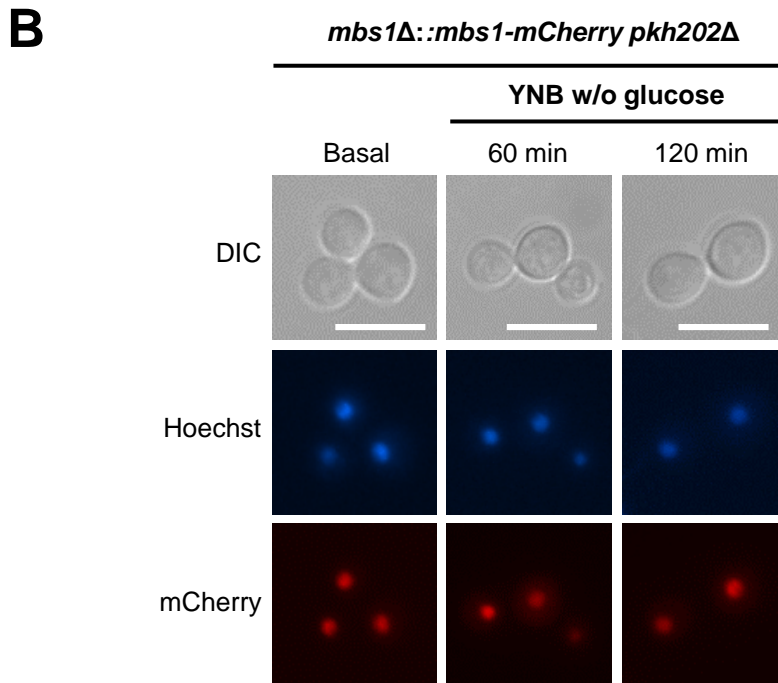

Supplement: FIG S3 [file mBio.02267-19-sf003.pdf]

Figure S4 (Lee et al.)

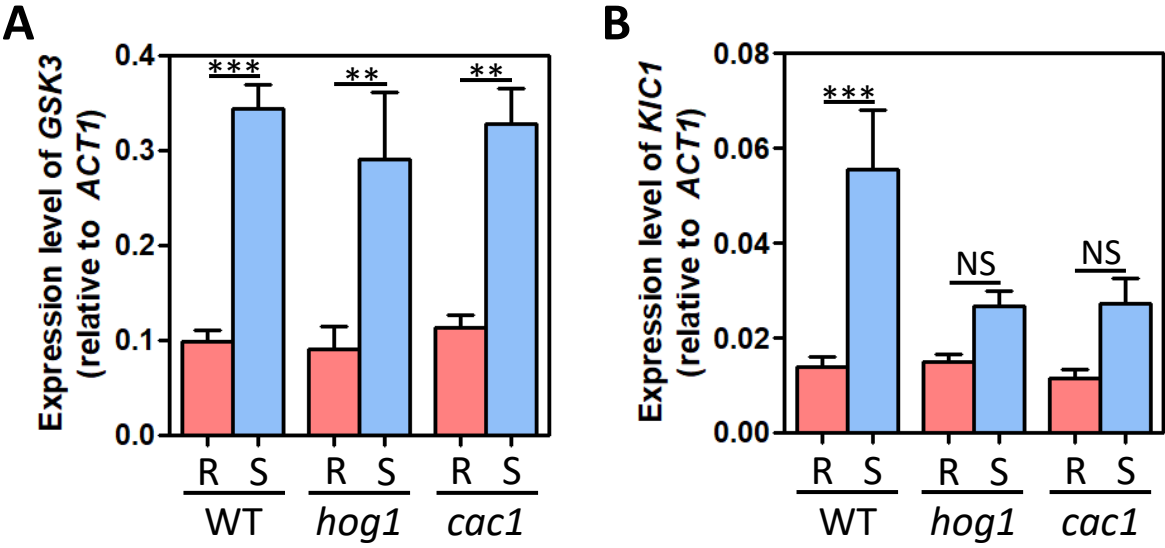

Supplement: FIG S4 [file mBio.02267-19-sf004.pdf]

Figure S5 (Lee et al.)

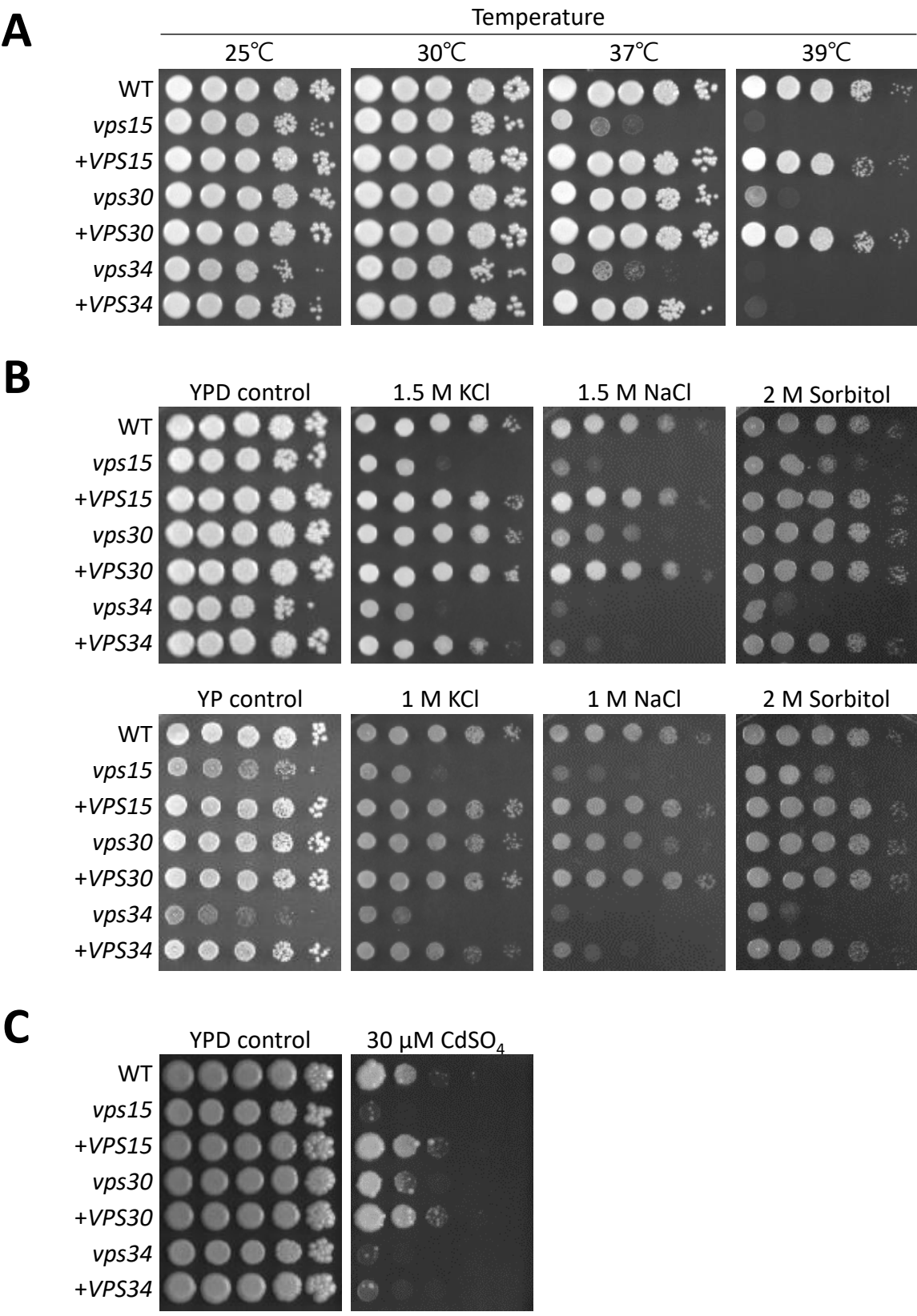

Continued

**D**

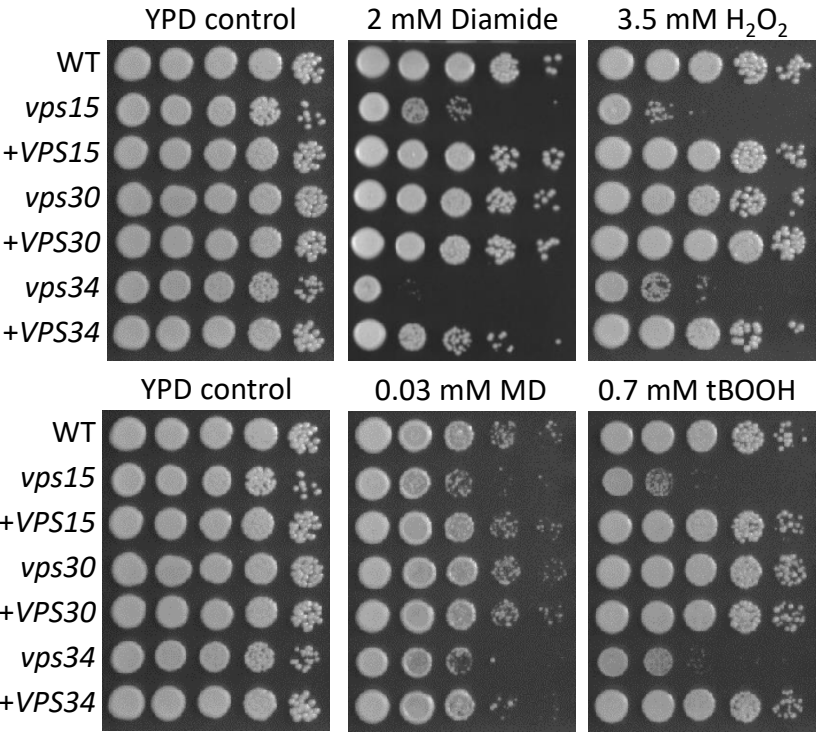

**E**

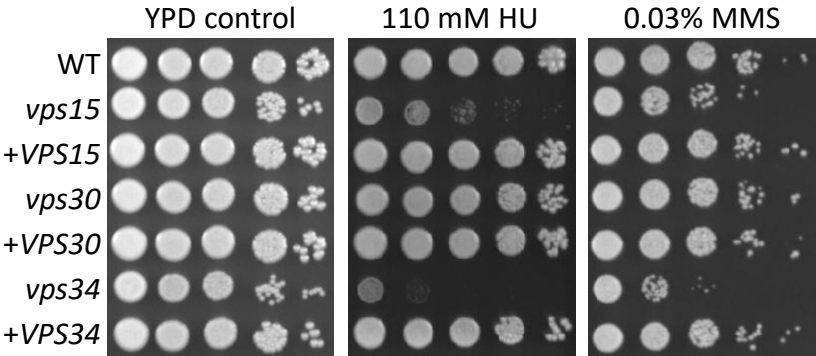

**F**

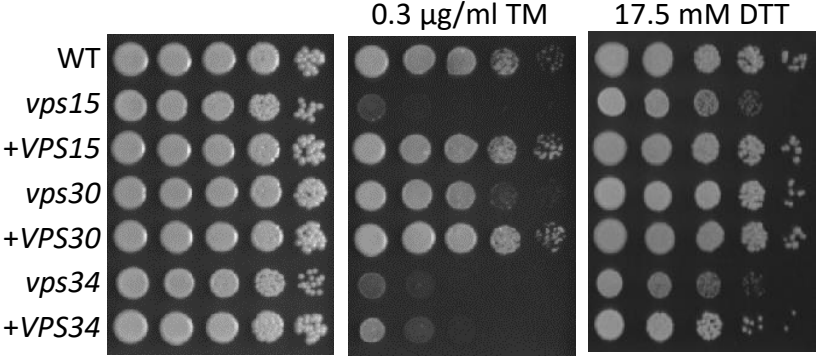

**G**

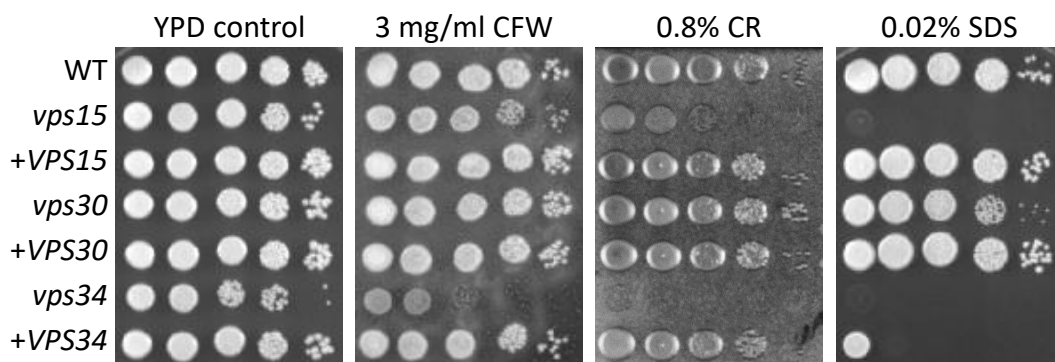

**H**

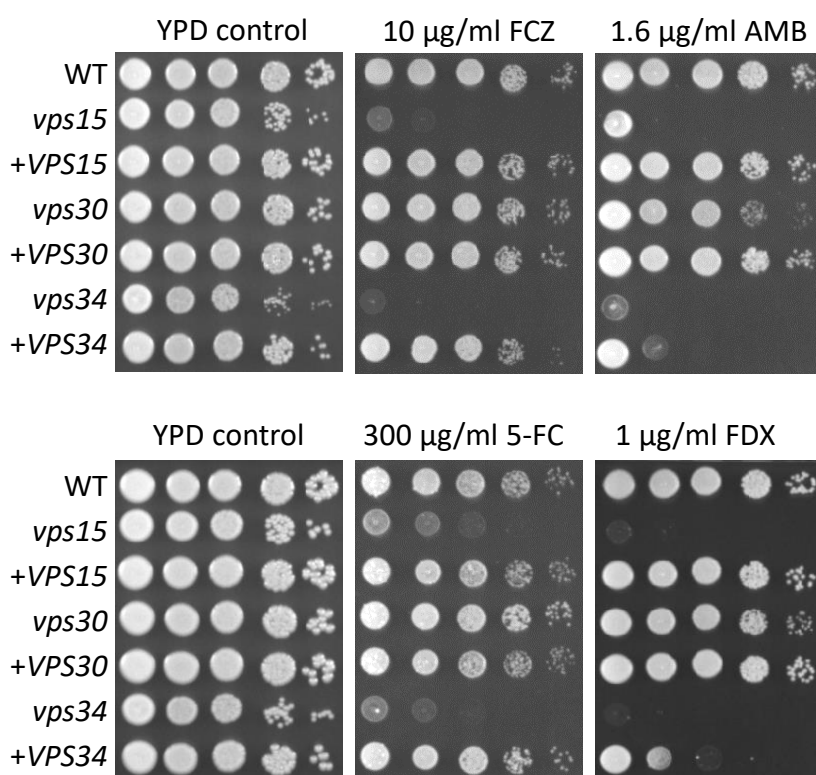

Supplement: FIG S5 [file mBio.02267-19-sf005.pdf]

Figure S6 (Lee et al.)

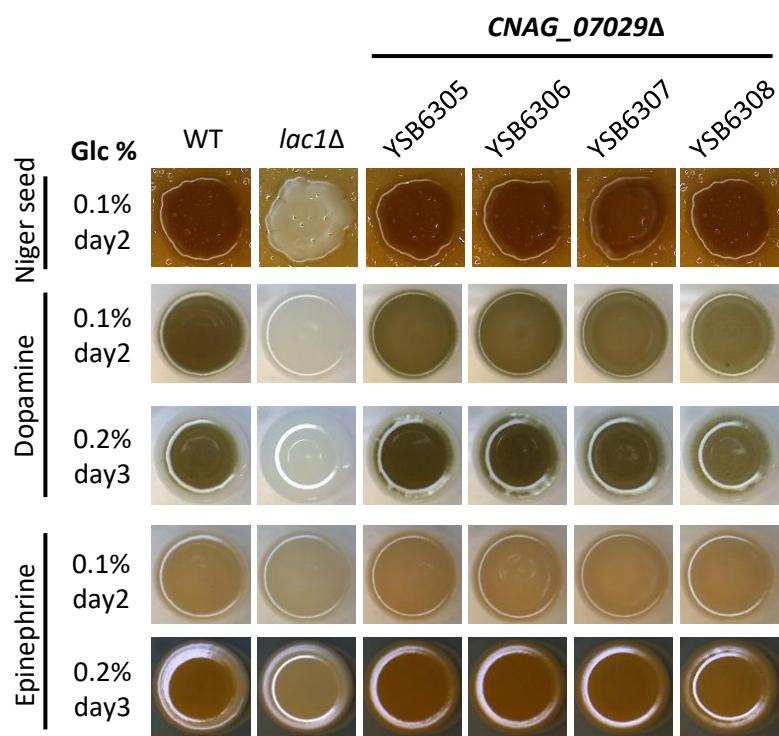

Supplement: FIG S6 [file mBio.02267-19-sf006.pdf]
