# Supplementary material for: Unraveling Melanin Biosynthesis and Signaling Networks in Cryptococcus neoformans
Source: mBio. 2019 Oct 1;10(5):e02267-19. doi: 10.1128/mBio.02267-19 (PMC6775464; doi:10.1128/mBio.02267-19)
Supplement: FIG S2 [file mBio.02267-19-sf002.pdf]

Figure S2 (Lee et al.)

A

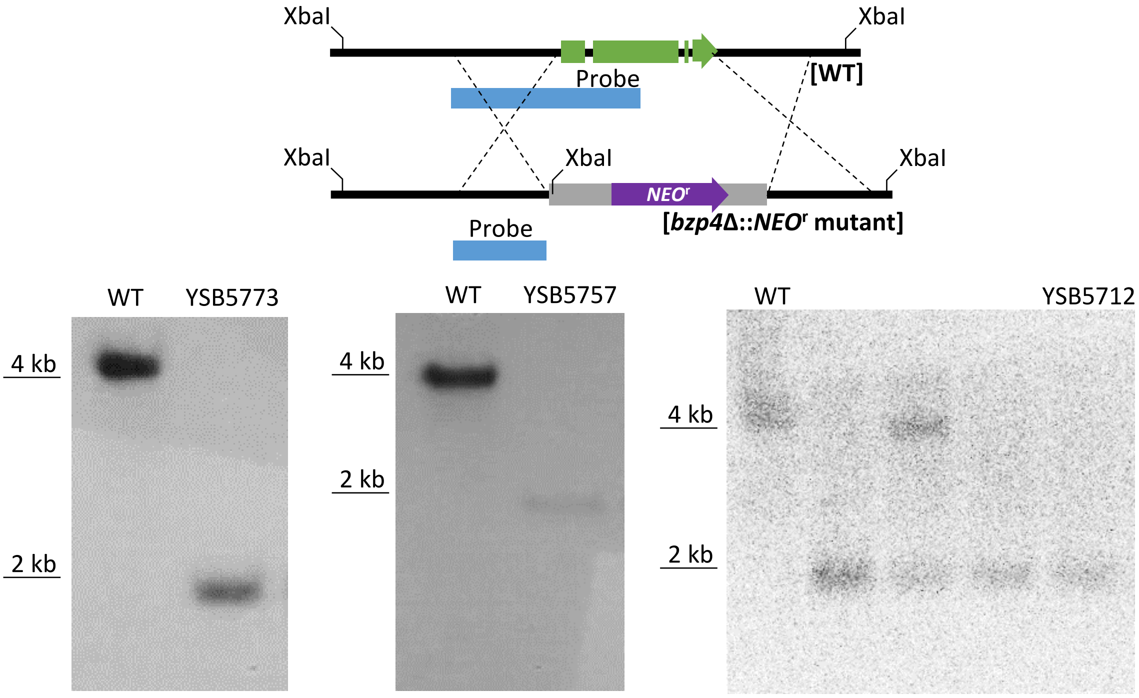

YSB5757 (*hob1Δ bzp4Δ*)
XbaI digestion
WT: 4444 bp
bzp4Δ::*NEO*ʳ: 1902 bp

YSB5757 (*usv101Δ bzp4Δ*)
XbaI digestion
WT: 4444 bp
bzp4Δ::*NEO*ʳ: 1902 bp

YSB5712 (*mbs1Δ bzp4Δ*)
XbaI digestion
WT: 4444 bp
bzp4Δ::*NEO*ʳ: 1902 bp

B

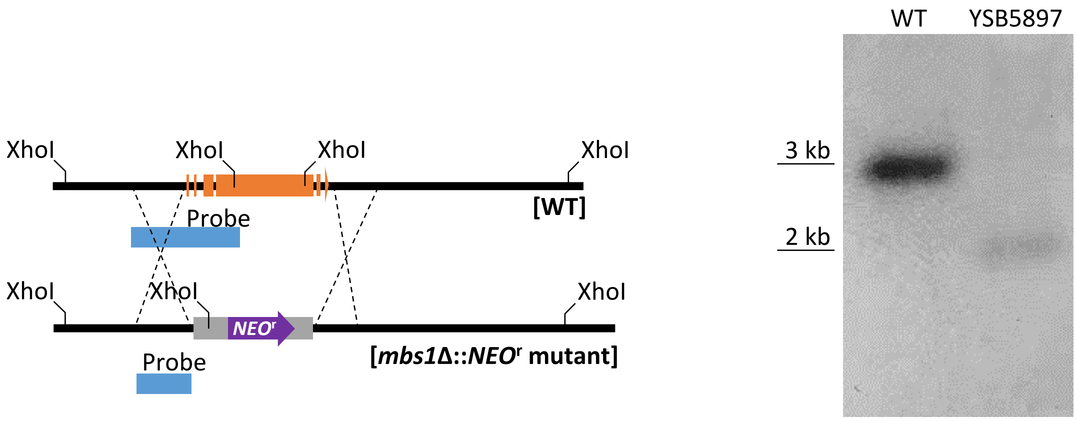

YSB5897 (*hob1Δ mbs1Δ*)
XhoI digestion
WT: 3130 bp
mbs1Δ::*NEO*ʳ: 2273 bp

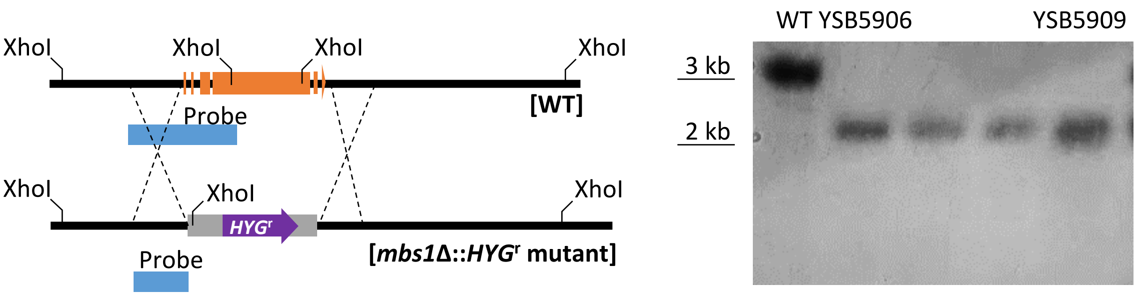

YSB5906 (*usv101Δ* bzp4Δ *mbs1Δ*)
YSB5909 (*usv101Δ mbs1Δ*)
XhoI digestion
WT: 3130 bp
YSB5906 (*mbs1Δ*::*HYG*ʳ): 2273 bp
YSB5909 (*mbs1Δ*::*NEO*ʳ): 2288 bp

Continued

C

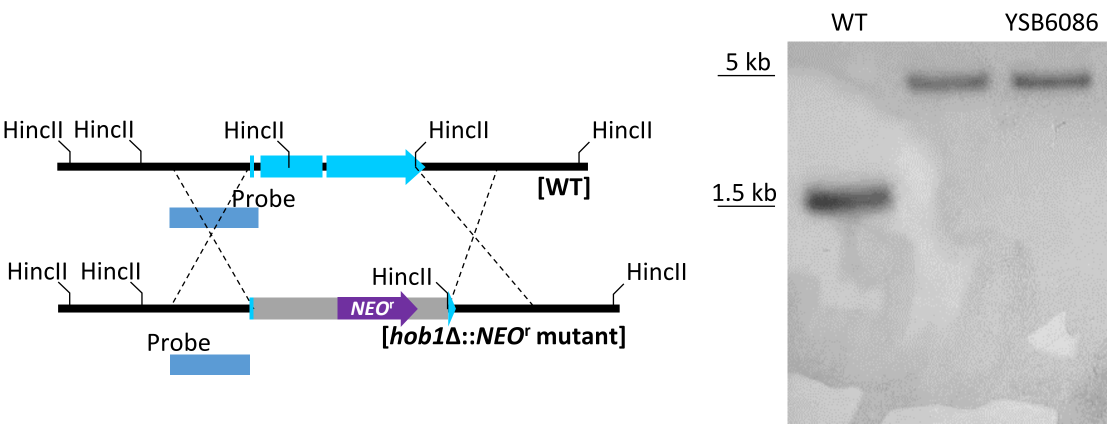

YSB6086 (*usv101Δ hob1Δ*)
HincII digestion
WT: 1583 bp
hob1Δ::NEO^r: 4975 bp

D

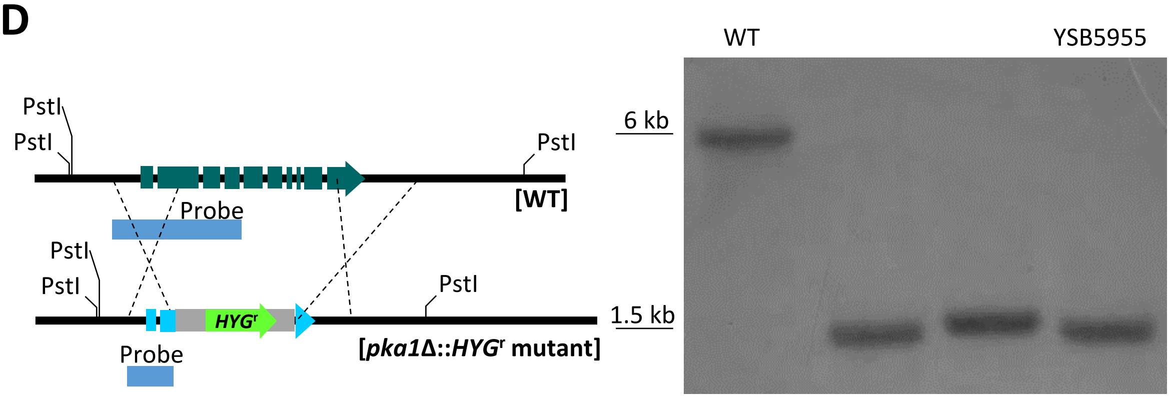

YSB5955 (*MBS1-mCherry pka1Δ*)
PstI digestion
WT: 6055 bp
pka1Δ::HYG^r: 1534 bp

E

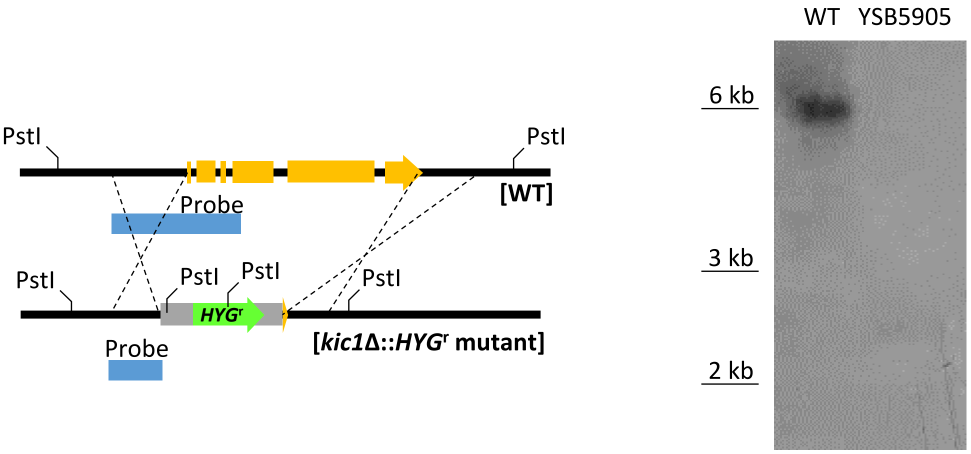

YSB5955 (*BZP4-mCherry kic1Δ*)
PstI digestion
WT: 6109 bp
kic1Δ::HYG^r: 2853 bp

Continued

F

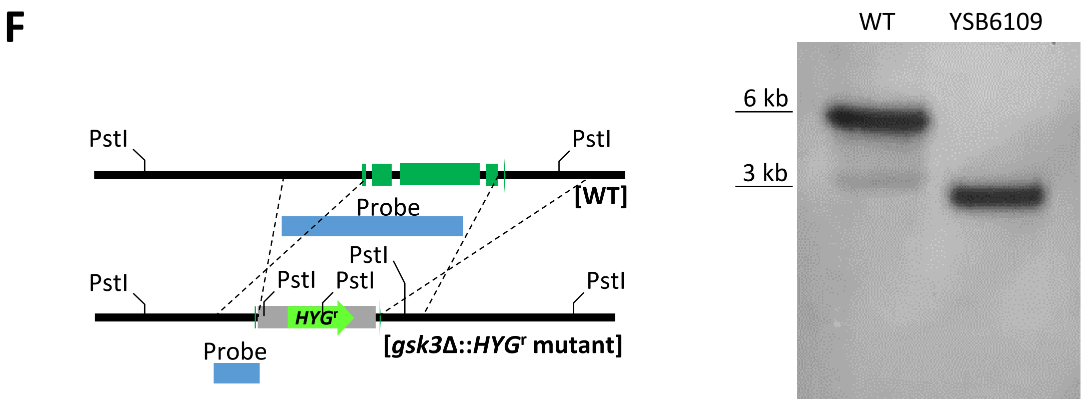

YSB6109 (*BZP4-mCherry gsk3Δ*)
PstI digestion
WT: 6109 bp
gsk3Δ::HYG^r: 2853 bp

G

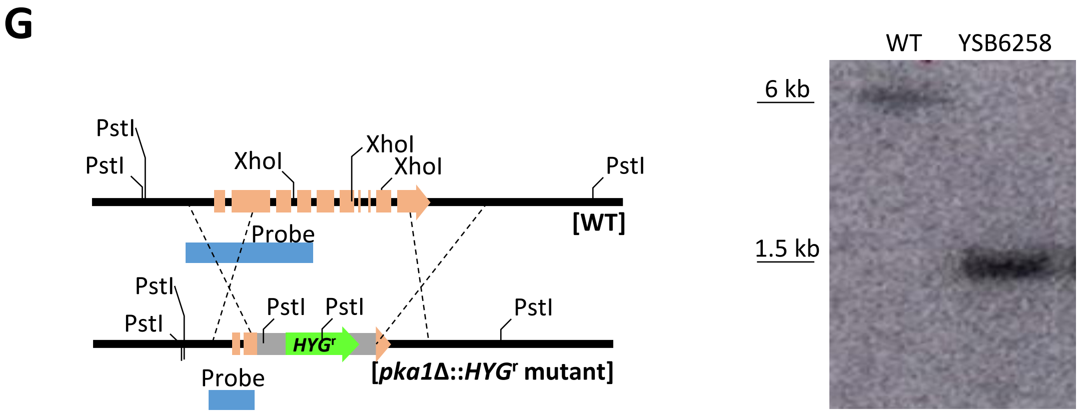

YSB6258 (*MBS1-mCherry pka1Δ*)
PstI digestion
WT: 6055 bp
pka1Δ::HYG^r: 1534 bp

H

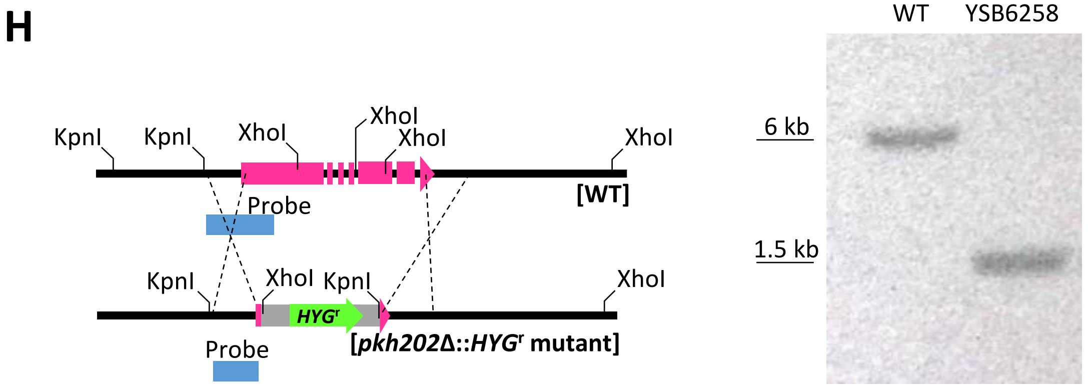

YSB6258 (*MBS1-mCherry pkh202Δ*)
XhoI and KpnI digestion
WT: 6055 bp
pka1Δ::HYG^r: 1534 bp

Continued

I

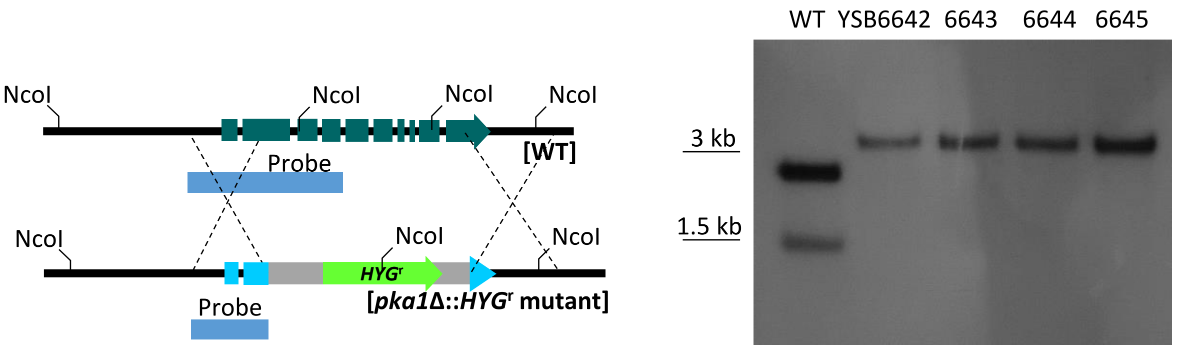

YSB (*USV101-mCherry pka1Δ*)
NcoI digestion
WT: 2632, 1443 bp
pka1Δ::HYG^r: 3654 bp

J

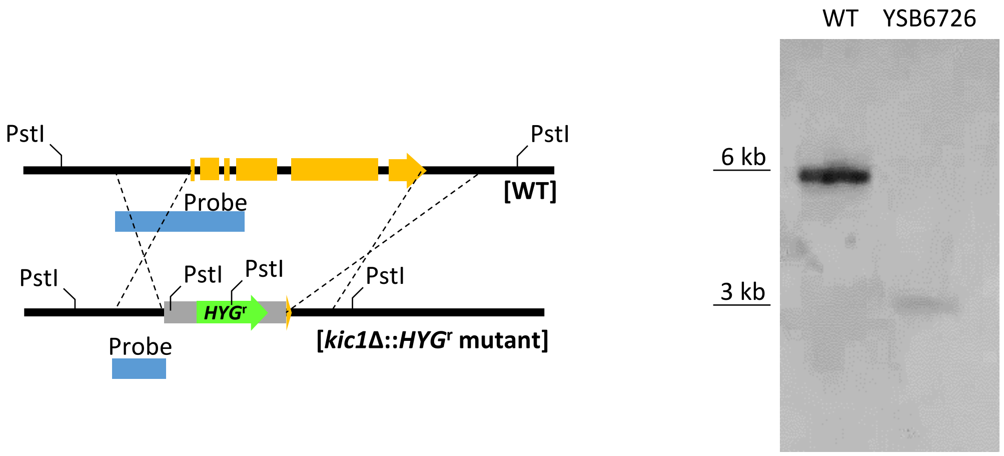

YSB6726 (*USV101-mCherry kic1Δ*)
PstI digestion
WT: 6109 bp
kic1Δ::HYG^r: 2853 bp

K

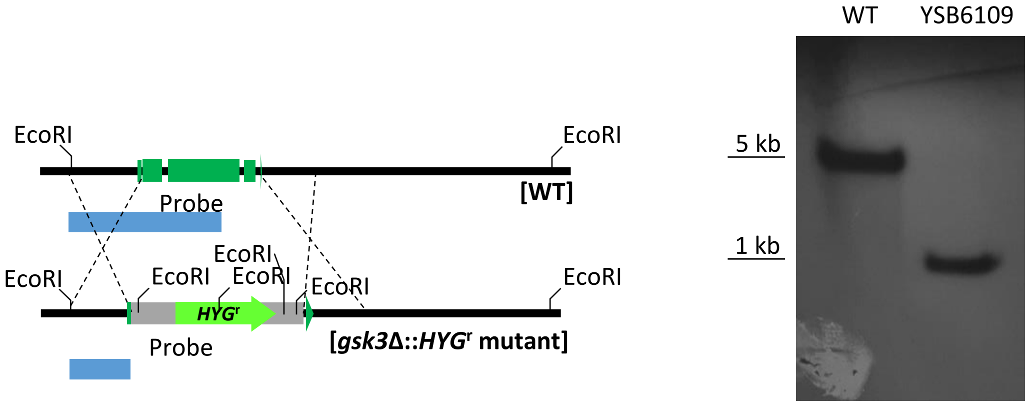

YSB6646 (*BZP4-mCherry gsk3Δ*)
EcoRI digestion
WT: 5401 bp
gsk3Δ::HYG^r: 893 bp

Continued

L

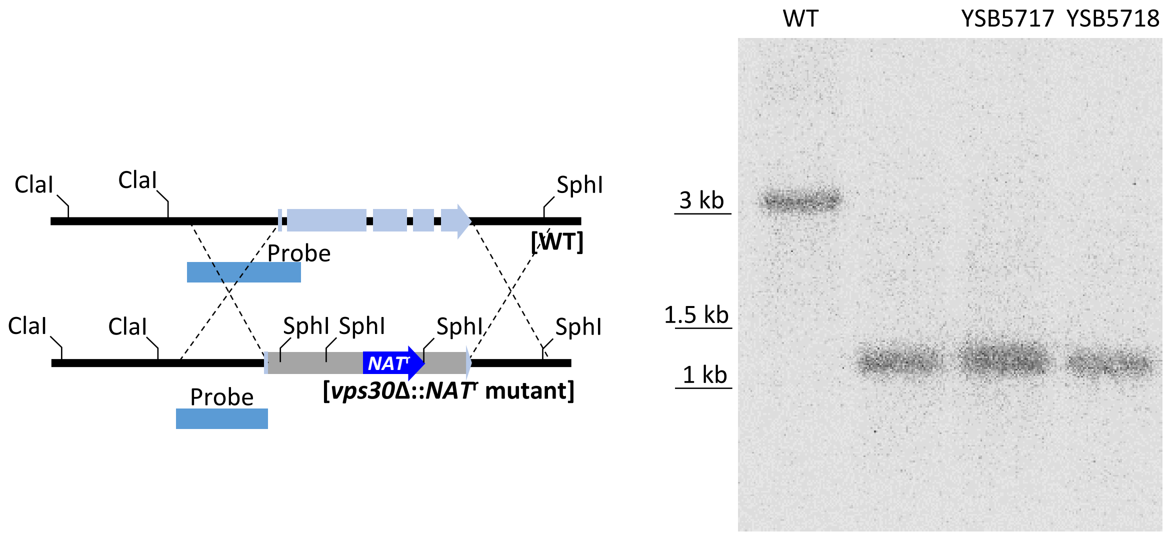

YSB5717 and YSB5718 (*vps30Δ*)
ClaI, SphI digestion
WT: 3501 bp
vps30Δ::NAT^r: 1204 bp

M

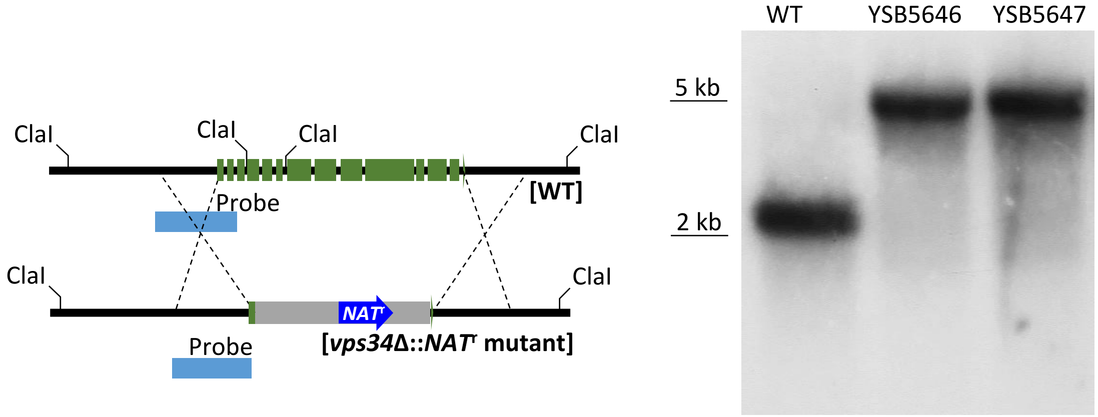

YSB5646 and YSB5647 (*vps34Δ*)
ClaI digestion
WT: 2384 bp
vps34Δ::NAT^r: 5325 bp

Continued

N

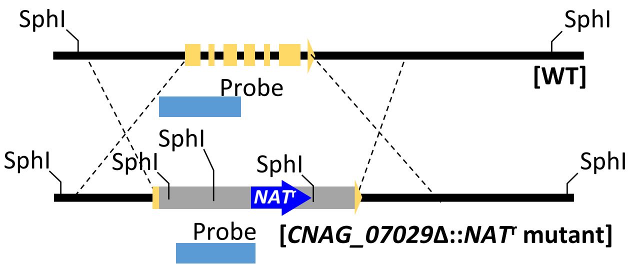

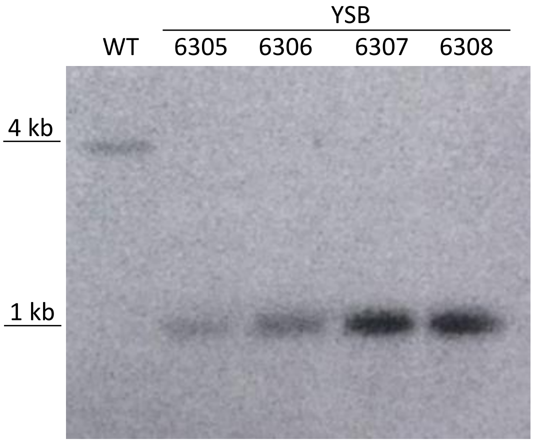

YSB6305, 6306, 6307, and 6308 (*CNAG_07029Δ*)
SphI digestion
WT: 3883 bp
CNAG_07029Δ::NAT^r: 1042 bp
