# Supplementary material for: Unraveling Melanin Biosynthesis and Signaling Networks in Cryptococcus neoformans
Source: mBio. 2019 Oct 1;10(5):e02267-19. doi: 10.1128/mBio.02267-19 (PMC6775464; doi:10.1128/mBio.02267-19)
Supplement: TABLE S1 [file mBio.02267-19-st001.docx]

**Table S1. *C. neoformans* strains used in this study**

| **Strain** | **Genotype** | **Parent** | **Reference** |
| --- | --- | --- | --- |
| H99 | *MAT*α |  | (1) |
| YSB42 | *MAT*α *cac1*Δ::*NAT-STM*#*159* | H99 | (2) |
| YSB64 | *MAT*α *hog1*Δ::*NAT-STM*#*177* | H99 | (3, 4) |
| CHM3 | *MAT*α *lac1*Δ::*NAT* | H99 | (2, 5) |
| YSB188 | *MAT*α *pka1*Δ::*NAT-STM#191* | H99 | (2, 4) |
| YSB488 | *MAT*α *mbs1*Δ::*NAT-STM*#*150* | H99 | (6) |
| YSB1465 | *MAT*α *usv101*Δ::*NAT-STM*#*191* | H99 | (6) |
| YSB1894 | *MAT*α *bzp4*Δ::*NAT-STM*#*295* | H99 | (6) |
| YSB2308 | *MAT*α *hob1*Δ::*NAT-STM#213* | H99 | (6) |
| YSB1500 | *MAT*α *vps15*Δ::*NAT-STM#123* | H99 | (4) |
| YSB2038 | *MAT*α *gsk3*Δ::*NAT-STM*#*123* | H99 | (4) |
| YSB2916 | *MAT*α *kic1*Δ::*NAT-STM#201* | H99 | (4) |
| YSB2942 | *MAT*α *cbk1*Δ::*NAT-STM#232* | H99 | (4) |
| YSB3063 | *MAT*α *mec1*Δ::*NAT-STM#204* | H99 | (4) |
| YSB3329 | *MAT*α *met3*Δ::*NAT-STM#169* | H99 | (4) |
| YSB3633 | *MAT*α *mps1*Δ::*NAT-STM#116* | H99 | (4) |
| YSB4268 | *MAT*α *pkh202*Δ::*NAT-STM#218* | H99 | (4) |
| YSB4270 | *MAT*α *pro1*Δ::*NAT-STM#5* | H99 | (4) |
| YSB1411 | *MAT*α *sks1*Δ::*NAT-STM#211* | H99 | (4) |
| YSB5712 | *MAT*α *mbs1*Δ::*NAT-STM#150* *bzp4*Δ::*NEO* | YSB488 | This study |
| YSB5757 | *MAT*α *usv101*Δ::*NAT-STM#191* *bzp4*Δ::*NEO* | YSB1465 | This study |
| YSB5773 | *MAT*α *hob1*Δ::*NAT-STM#213* *bzp4*Δ::*NEO* | YSB2309 | This study |
| YSB5897 | *MAT*α *hob1*Δ::*NAT-STM#213* *mbs1*Δ::*NEO* | YSB2309 | This study |
| YSB5909 | *MAT*α *usv101*Δ::*NAT-STM#191* *mbs1*Δ::*NEO* | YSB1465 | This study |
| YSB6086 | *MAT*α *usv101*Δ::*NAT-STM#191* *hob1*Δ::*NEO* | YSB1465 | This study |
| YSB5906 | *MAT*α *usv101*Δ::*NAT-STM#191* *bzp4*Δ::*NEO* *mbs1*Δ::*HYG* | YSB5757 | This study |
| YSB6293 | *MAT*α *usv101*Δ + *USV101*-*mCherry*-*NEO* | YSB1465 | This study |
| YSB6642 | *MAT*α *usv101*Δ + *USV101*-*mCherry*-*NEO* *pka1*Δ::*HYG* | YSB6293 | This study |
| YSB6726 | *MAT*α *usv101*Δ + *USV101*-*mCherry*-*NEO* *kic1*Δ::*HYG* | YSB6293 | This study |
| YSB6646 | *MAT*α *usv101*Δ + *USV101*-*mCherry*-*NEO* *gsk3*Δ::*HYG* | YSB6293 | This study |
| YSB5408 | *MAT*α *bzp4*Δ::*BZP4*-*mCherry*-*NEO* | YSB1895 | This study |
| YSB5955 | *MAT*α *bzp4*Δ::*BZP4*-*mCherry*-*NEO* *pka1*Δ::*HYG* | YSB5408 | This study |
| YSB5905 | *MAT*α *bzp4*Δ::*BZP4-mCherry-NEO* *kic1*Δ::*HYG* | YSB5408 | This study |
| YSB6109 | *MAT*α *bzp4*Δ::*BZP4-mCherry-NEO* *gsk3*Δ::*HYG* | YSB5408 | This study |
| YSB4766 | *MAT*α *mbs1*Δ::*MBS1*-*mCherry-NEO* | YSB489 | This study |
| YSB6258 | *MAT*α *mbs1*Δ::*MBS1-mCherry-NEO* *pka1*Δ::*HYG* | YSB4766 | This study |
| YSB6383 | *MAT*α *mbs1*Δ::*MBS1-mCherry-NEO* *pkh202*Δ::*HYG* | YSB4766 | This study |
| YSB6305 | *MAT*α CNAG_07029Δ::*NAT-STM#240* | H99 | This study |
| YSB6306 | *MAT*α CNAG_07029Δ::*NAT-STM#240* | H99 | This study |
| YSB6307 | *MAT*α CNAG_07029Δ::*NAT-STM#240* | H99 | This study |
| YSB6308 | *MAT*α CNAG_07029Δ::*NAT-STM#240* | H99 | This study |
| YSB5509 | *MAT*α *vps15*Δ::*VPS15-mCherry-NEO* | YSB1500 | This study |
| YSB5721 | *MAT*α *vps30*Δ::*NAT-STM#123* | H99 | This study |
| YSB6191 | *MAT*α *vps30*Δ::*VPS30-mCherry-NEO* | YSB5721 | This study |
| YSB5646 | *MAT*α *vps34*Δ::*NAT-STM#123* | H99 | This study |
| YSB6555 | *MAT*α *vps34*Δ::*VPS34-mCherry-NEO* | YSB6555 | This study |

**References**

1. Perfect JR, Ketabchi N, Cox GM, Ingram CW, Beiser CL. 1993. Karyotyping of *Cryptococcus neoformans* as an epidemiological tool. J Clin Microbiol 31:3305-9.

2. Bahn YS, Hicks JK, Giles SS, Cox GM, Heitman J. 2004. Adenylyl cyclase-associated protein Aca1 regulates virulence and differentiation of *Cryptococcus neoformans* via the cyclic AMP-protein kinase A cascade. Eukaryot Cell 3:1476-91.

3. Bahn YS, Kojima K, Cox GM, Heitman J. 2005. Specialization of the HOG pathway and its impact on differentiation and virulence of *Cryptococcus neoformans*. Mol Biol Cell 16:2285-300.

4. Lee KT, So YS, Yang DH, Jung KW, Choi J, Lee DG, Kwon H, Jang J, Wang LL, Cha S, Meyers GL, Jeong E, Jin JH, Lee Y, Hong J, Bang S, Ji JH, Park G, Byun HJ, Park SW, Park YM, Adedoyin G, Kim T, Averette AF, Choi JS, Heitman J, Cheong E, Lee YH, Bahn YS. 2016. Systematic functional analysis of kinases in the fungal pathogen *Cryptococcus neoformans*. Nat Commun 7:12766.

5. Hicks JK, D'Souza CA, Cox GM, Heitman J. 2004. Cyclic AMP-dependent protein kinase catalytic subunits have divergent roles in virulence factor production in two varieties of the fungal pathogen *Cryptococcus neoformans*. Eukaryotic Cell 3:14-26.

6. Jung KW, Yang DH, Maeng S, Lee KT, So YS, Hong J, Choi J, Byun HJ, Kim H, Bang S, Song MH, Lee JW, Kim MS, Kim SY, Ji JH, Park G, Kwon H, Cha S, Meyers GL, Wang LL, Jang J, Janbon G, Adedoyin G, Kim T, Averette AK, Heitman J, Cheong E, Lee YH, Lee YW, Bahn YS. 2015. Systematic functional profiling of transcription factor networks in *Cryptococcus neoformans*. Nat Commun 6:6757.
