# Supplementary material for: Unraveling Melanin Biosynthesis and Signaling Networks in Cryptococcus neoformans
Source: mBio. 2019 Oct 1;10(5):e02267-19. doi: 10.1128/mBio.02267-19 (PMC6775464; doi:10.1128/mBio.02267-19)
Supplement: TABLE S2 [file mBio.02267-19-st002.docx]

**Table S2. Primers used in this study**

| Name | Primer description | Sequence (5’ to 3’) |
| --- | --- | --- |
| B79 | Common diagnostic screening primer | TGTGGATGCTGGCGGAGGATA |
| B1886 | *NEO* split primer 1 | TGGAAGAGATGGATGTGC |
| B1887 | *NEO* split primer 2 | ATTGTCTGTTGTGCCCAG |
| B5751 | *HYG* split primer 1 | CGAAGAATCTCGTGCTTTC |
| B5752 | *HYG* split primer 2 | ATTGACCGATTCCTTGCG |
| B1026 | M13 forward-extended primer | GTAAAACGACGGCCAGTGAGC |
| B1027 | M13 reverse-extended primer | CAGGAAACAGCTATGACCATG |
| B3738 | *BZP4* 5’-flanking region primer 1 | CGCCCTTTCTATTGTTACAC |
| B3739 | *BZP4* 5’-flanking region primer 2 | TCACTGGCCGTCGTTTTACGATGACAGGAGGGATGAATC |
| B3740 | *BZP4* 3’-flanking region primer 1 | CATGGTCATAGCTGTTTCCTGGGGAGAATAACGACTCAATGTC |
| B3741 | *BZP4* 3’-flanking region primer 2 | TCATTGCTGACTGGGAAG |
| B3736 | *BZP4* diagnostic screening primer, pairing with B79 | AAAGAGGCGGTGTTGAAG |
| B3737 | *BZP4* Southern blot probe primer, paring with B3738 | AGCCAGGTAATCTTGGAGG |
| B9782 | CNAG_07029 5’-flanking region primer 1 | TTTCCCTCTGGTGCTATTC |
| B9783 | CNAG_07029 5’-flanking region primer 2 | TCACTGGCCGTCGTTTTACTGTGGCTGTGTTGCTGTTG |
| B9784 | CNAG_07029 3’-flanking region primer 1 | CATGGTCATAGCTGTTTCCTGCTCTTCATCGCTCAGTTCTG |
| B9785 | CNAG_07029 3’-flanking region primer 2 | CTGCCCTCTTAGTCATTCG |
| B9786 | CNAG_07029 diagnostic screening primer, pairing with B79 | GATGTATTGTGTGTGGGGAC |
| B9787 | CNAG_07029 Southern blot probe primer, paring with B9782 | TGAGAGGATTGTTATCTGGG |
| B4431 | *GSK3* 5’-flanking region primer 1 | GTGAGTCTATCCTTCGTTTCTGTC |
| B4432 | *GSK3* 5’-flanking region primer 2 | TCACTGGCCGTCGTTTTACCGGCTTCCAAAAAAGTCAG |
| B4433 | *GSK3* 3’-flanking region primer 1 | CATGGTCATAGCTGTTTCCTGCTGAACAACTGCGTGTCAC |
| B4434 | *GSK3* 3’-flanking region primer 2 | CTTGAAAGATGACGCTCG |
| B4435 | *GSK3* diagnostic screening primer, pairing with B79 | ACATCCTTTGTCTCCCCCAC |
| B4436 | *GSK3* Southern blot probe primer, paring with B4431 | CGGAAGACTTTGGTGAAGG |
| B7124 | *HOB1* 5’-flanking region primer 1 | CACTTCTAACCCTGAATACTGC |
| B2800 | *HOB1* 5’-flanking region primer 2 | TCACTGGCCGTCGTTTTACTGTCTTCGTTCTTGCCCTCC |
| B2801 | *HOB1* 3’-flanking region primer 1 | CATGGTCATAGCTGTTTCCTGCGTTGACAGAAGAGGACAAGG |
| B2802 | *HOB1* 3’-flanking region primer 2 | TGACAGATGATGAGCAGAGG |
| B2798 | *HOB1* diagnostic screening primer, pairing with B79 | ATTCCAGAGCCTCACTTGC |
| B2803 | *HOB1* Southern blot probe primer, paring with B7124 | TGTATGAGGTCTTGTCCACC |
| B3168 | *KIC1* 5’-flanking region primer 1 | AAGATGAGCGTTGCGAAG |
| B3169 | *KIC1* 5’-flanking region primer 2 | TCACTGGCCGTCGTTTTACGCGTGGTGCTAAGAACAAC |
| B3170 | *KIC1* 3’-flanking region primer 1 | CATGGTCATAGCTGTTTCCTGGAGGTAGACTCCCAGAATGC |
| B3171 | *KIC1* 3’-flanking region primer 2 | TAATGTGTCAACTGCCGC |
| B3166 | *KIC1* diagnostic screening primer, pairing with B79 | TTGGTTTCAAGGGGGAAC |
| B3167 | *KIC1* Southern blot probe primer, paring with B3168 | AAAGTGGACCGTTTGGAG |
| B1212 | *MBS1* 5’-flanking region primer 1 | GGCATCAGGATAGAAACGC |
| B1213 | *MBS1* 5’-flanking region primer 2 | GCTCACTGGCCGTCGTTTTACGGGGTGAGATTTGAAGGTAG |
| B1214 | *MBS1* 3’-flanking region primer 1 | CATGGTCATAGCTGTTTCCTGGATGTGACTTGGTCTTGGG |
| B1215 | *MBS1* 3’-flanking region primer 2 | TGGTCCAGTCTCCTCATTAC |
| B1216 | *MBS1* diagnostic screening primer, pairing with B79 | TCTTCCATCTCAGCATCG |
| B4927 | *MBS1* Southern blot probe primer, paring with B1212 | ATTTTGGGACAGCGTCAC |
| JOHE12911 | *PKA1* 5’-flanking region primer 1 | AAACGACTGTGTAATGCGAG |
| JOHE12912 | *PKA1* 5’-flanking region primer 2 | CTGGCCGTCGTTTTACGGAGCCAGAATAAAGGAGTTG |
| JOHE12935 | *PKA1* 3’-flanking region primer 1 | GTCATAGCTGTTTCCTGGCACTAAATGGGTGAGCAC |
| JOHE12937 | *PKA1* 3’-flanking region primer 2 | CGATTTGTCCAGTGATTCAGTGAC |
| JOHE12915 | *PKA1* diagnostic screening primer, pairing with B79 | GTTGGAAGTAGCAGTGTCTTG |
| B7368 | *PKA1* Southern blot probe primer, paring with BJOHE12911 | TGTCGGAGGAGAATGAACG |
| B3210 | *PKH202* 5’-flanking region primer 1 | TGGTGGAAATGGACTGTG |
| B3211 | *PKH202* 5’-flanking region primer 2 | TCACTGGCCGTCGTTTTACCAGCCTCGGGTTTTTTTG |
| B3212 | *PKH202* 3’-flanking region primer 1 | CATGGTCATAGCTGTTTCCTGAGCACGAAAAGCACGAAG |
| B3213 | *PKH202* 3’-flanking region primer 2 | AACTGGTAGCCATTCTGGG |
| B3208 | *PKH202* diagnostic screening primer, pairing with B79 | AGGTGGGATTGCTCAAAC |
| B3209 | *PKH202* Southern blot probe primer, , paring with B3210 | TGAAGGCGTGCTCAAATG |
| B9332 | *VPS30* 5’-flanking region primer 1 | AACGAGGGTTGAGCAGATGG |
| B9333 | *VPS30* 5’-flanking region primer 2 | TCACTGGCCGTCGTTTTAC ACCTGGTTACATCTCTGGC |
| B9334 | *VPS30* 3’-flanking region primer 1 | CATGGTCATAGCTGTTTCCTGGGGTCCTCATAATGGAGAGTG |
| B9335 | *VPS30* 3’-flanking region primer 2 | CTCAGGTAACTGGAGTGAAC |
| B9336 | *VPS30* diagnostic screening primer, pairing with B79 | CCGAGGGGAAGATGAATAATC |
| B9337 | *VPS30* Southern blot probe primer, , paring with B9332 | CGGAGAGTTTATGCCTTGG |
| B9277 | *VPS34* 5’-flanking region primer 1 | ACAAAATCGTCACCAGGAAG |
| B9278 | *VPS34* 5’-flanking region primer 2 | TCACTGGCCGTCGTTTTACCTCGCAAAGGAATAGTCCC |
| B9279 | *VPS34* 3’-flanking region primer 1 | CATGGTCATAGCTGTTTCCTGGCAGTATCTTCGGGATTAGAC |
| B9280 | *VPS34* 3’-flanking region primer 2 | TGTAATGATGACGGACACG |
| B9281 | *VPS34* diagnostic screening primer, pairing with B79 | CTCATACTTGTGCTCCTTGG |
| B9282 | *VPS34* Southern blot probe primer, , paring with B9277 | TTCTGTGGAGGGTGAGAGTTGG |
| B9003 | LP for *BZP4* complementation | CTCGAGCGCTTTCGCAATGTCAGG |
| B9004 | RP for *BZP4* complementation | GCGGCCGCTCTTGACATTGAGTCGTT |
| B9032 | Screening primer for *BZP4* complementation | AGAGACTACAGCAGCCAAG |
| B9005 | *BZP4* sequencing primer 1 | GTTACTGTTACAGCGAAC |
| B1958 | LP for *MBS1* complementation | CGCGCGGCCGCGGCATCAGGATAGAAACGC |
| B8329 | RP for *MBS1* complementation | GCGGCCGCCTGCACCCATGATGAAGG |
| B8043 | Screening primer for *MBS1* complementation | GGACCTACCATCCCAAAGAG |
| B1960 | *MBS1* sequencing primer 1 | GTATTCCGCAAAACATCCTC |
| B1961 | *MBS1* sequencing primer 2 | TGGTGTTGAAGACATTCTCC |
| B1962 | *MBS1* sequencing primer 3 | ATGCCGAACAAACTCCTC |
| B1963 | *MBS1* sequencing primer 4 | TTGAGGGCGAACTGATAAG |
| B9494 | LP for *USV101* complementation | TCTAGAACACATCCTGTCTTGCTGTGG |
| B9495 | RP for *USV101* complementation | GCGGCCGCCTGCGTCTTCTTATCATCC |
| B9499 | Screening primer for *USV101* complementation | CCCCCAAATCACAAATCC |
| B9496 | *USV101* sequencing primer 1 | ATCGGACTTTCTTGCGAC |
| B9497 | *USV101* sequencing primer 2 | TCCCATAGTTATCCTCCTCAG |
| B9498 | *USV101* sequencing primer 3 | TCAATGACGATGCTGGAC |
| B8722 | LP1 for *VPS15* complementation | GCGGCCGCGCGGTTGCGGCCTTAACTTG |
| B8723 | RP1 for *VPS15* complementation | GCTAGCGGGGGGATATCC |
| B8724 | LP2 for *VPS15* complementation | GCTAGCATCATCGACCAAG |
| B8725 | RP2 for *VPS15* complementation | GCGGCCGCACGCAAGGGACCAGCCATCC |
|  | Screening primer for *VPS15* complementation | CCTTTACCATCCAACCAACG |
| B8877 | *VPS15* sequencing primer 1 | GCCCGTCAGAGTCCAATC |
| B8878 | *VPS15* sequencing primer 2 | CGAGGTATTCAAAGGCAG |
| B8879 | *VPS15* sequencing primer 3 | CACCCTCTTTGCCTTCGC |
| B9526 | LP for *VPS30* complementation | GCGGCCGCACTCTGTTCATTTATATT |
| B9527 | RP for *VPS30* complementation | GGGCCCTTGACTGGGATGAGGACTG |
| B9528 | Screening primer for *VPS30* complementation | CCCACAAATCCTTTCTCAC |
| B9529 | *VPS30* sequencing primer 1 | ACCAATGACTCTTTCCACG |
| B9530 | *VPS30* sequencing primer 2 | TCCTACTCTCAGATTCCGC |
| B9531 | *VPS30* sequencing primer 3 | TGTTCCGCTTTTACCTCTC |
| B9512 | LP for *VPS34* complementation | GCGGCCGCATCCCGAAGATACTGCGCC |
| B9513 | RP for *VPS34* complementation | GGGCCCGCCATAACTTCCTGTTTGG |
| B9512 | Screening primer for *VPS34* complementation | TCCATCTCCAATGACTTCC |
| B9513 | *VPS34* sequencing primer 1 | ACACCCTTGAGATTCCTCC |
| B9514 | *VPS34* sequencing primer 2 | CGACGGCAAACAATACTC |
| B9515 | *VPS34* sequencing primer 3 | TTCCCTGTAGTCTTCTCGG |
| B9516 | *VPS34* sequencing primer 4 | ATCGTAGCCATCGCAAAC |
| B9517 | *VPS34* sequencing primer 5 | ATCGCCTTCTACGCAAAG |
| B679 | *ACT1* qRT-PCR primer 1 | CGCCCTTGCTCCTTCTTCTATG |
| B680 | *ACT1* qRT-PCR primer 2 | GACTCGTCGTATTCGCTCTTCG |
| B8953 | LAC1 qRT-PCR primer 1 | CACCCTTTGGAAGTTGTGG |
| B8954 | LAC1 qRT-PCR primer 2 | TGATAATTGCAGAGTACCG |
| B6376 | *USV101* qRT-PCR primer 1 | CGAATCCCAGTGGAATGCC |
| B4995 | *USV101* qRT-PCR primer 2 | TCCAGCATCGTCATTGAG |
| B2313 | *MBS1* qRT-PCR primer 1 | TATCACCAATCAAGCGGC |
| B2314 | *MBS1* qRT-PCR primer 2 | TCTCCTCTTCGTTTCCCTCG |
| B6394 | *HOB1* qRT-PCR primer 1 | CCTCGCAAGTTCCCCAGCTA |
| B8519 | *HOB1* qRT-PCR primer 2 | GTATGAGGTCTTGTCCACC |
| B8521 | *BZP4* qRT-PCR primer paring with B3737 | CAATATACGAATCACTCCC |
